# Distinct Molecular Trajectories Converge to Induce Naive Pluripotency

**DOI:** 10.1016/j.stem.2019.07.009

**Published:** 2019-09-05

**Authors:** Hannah T. Stuart, Giuliano G. Stirparo, Tim Lohoff, Lawrence E. Bates, Masaki Kinoshita, Chee Y. Lim, Elsa J. Sousa, Katsiaryna Maskalenka, Aliaksandra Radzisheuskaya, Andrew A. Malcolm, Mariana R.P. Alves, Rebecca L. Lloyd, Sonia Nestorowa, Peter Humphreys, William Mansfield, Wolf Reik, Paul Bertone, Jennifer Nichols, Berthold Göttgens, José C.R. Silva

**Affiliations:** 1 Wellcome-MRC Cambridge Stem Cell Institute, University of Cambridge, Cambridge, UK; 2 Department of Biochemistry, University of Cambridge, Cambridge, UK; 3 Department of Physiology, Development and Neuroscience, University of Cambridge, Cambridge, UK; 4 Department of Haematology and Cambridge Institute for Medical Research, University of Cambridge, Cambridge, UK; 5 Babraham Institute and Wellcome Sanger Institute, Cambridge, UK

**Keywords:** cell identity transitions, reprogramming, pluripotency, transcriptional networks, signaling

## Abstract

Understanding how cell identity transitions occur and whether there are multiple paths between the same beginning and end states are questions of wide interest. Here we show that acquisition of naive pluripotency can follow transcriptionally and mechanistically distinct routes. Starting from post-implantation epiblast stem cells (EpiSCs), one route advances through a mesodermal state prior to naive pluripotency induction, whereas another transiently resembles the early inner cell mass and correspondingly gains greater developmental potency. These routes utilize distinct signaling networks and transcription factors but subsequently converge on the same naive endpoint, showing surprising flexibility in mechanisms underlying identity transitions and suggesting that naive pluripotency is a multidimensional attractor state. These route differences are reconciled by precise expression of Oct4 as a unifying, essential, and sufficient feature. We propose that fine-tuned regulation of this “transition factor” underpins multidimensional access to naive pluripotency, offering a conceptual framework for understanding cell identity transitions.

## Highlights

• Reprogramming routes differ transcriptionally and mechanistically
• Reprogramming intermediates resemble different developmental stages
• Distinct routes converge on precise Oct4 regulation to permit identity transition
• Precise Oct4 expression is sufficient for reprogramming of EpiSCs and fibroblasts

## Graphical Abstract

**Figure:**
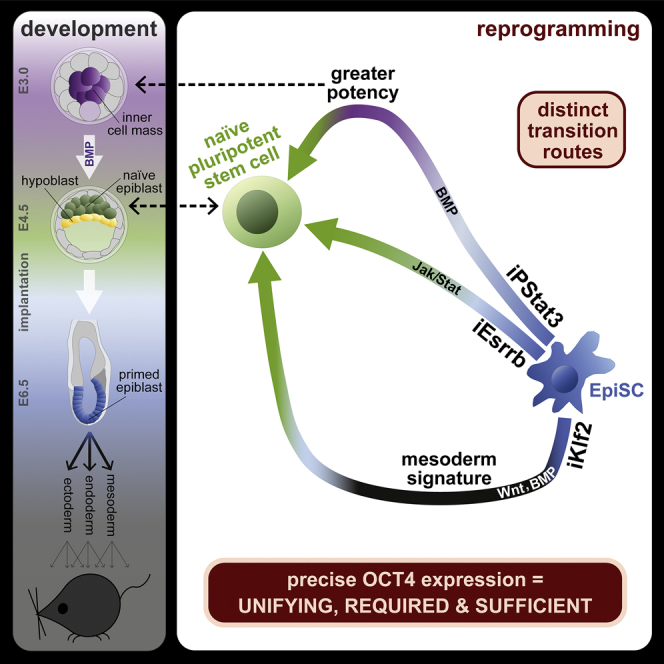

## Teaser

Stuart et al. report distinct routes of reprogramming to naive pluripotency. These differ in their transcriptional trajectories, mechanistic requirements, and developmental parallels, thus demonstrating considerable flexibility for a given cell identity transition to occur. Distinct routes converge on precise Oct4 expression, which is necessary and sufficient for naive pluripotency induction.

## Introduction

Differential use of the same genome generates the spectacular diversity of form and function in multicellular animals. Finite numbers of transcription factors (TFs) and signaling pathways are used in different combinations and contexts to generate this array of distinct cellular identities. But how is interplay between external signals and internal TF networks computed by the cell to instruct identity? Are there multiple routes by which a given identity can be established, or must it always follow the same progression of mechanistic steps? These are fundamental questions of wide interest, and the answers will underpin our understanding of multicellular biology.

A cellular identity with a stable gene regulatory network can be thought of as an attractor, occupying a local minimum in an “energetic” landscape of cell states (Kauffman, 1993; reviewed in Enver et al., 2009). But is an attractor multidimensional, with multiple ways by which it can be approached, or do identity transitions follow a set path through an energetic “valley”? Empirical evidence supporting theories of cellular identity as a multidimensional attractor was provided in a landmark work by Huang et al. (2005). They showed two transcriptionally distinct routes of promyelocytic HL60 cell differentiation into neutrophils, although they noted some disparity in the resulting neutrophil identities. A limitation for further understanding the principles governing cell identity change has been a lack of suitable *in vitro* cell types and of defined, tractable systems to study the transitions occurring between these.

Here we investigate the principles underpinning cell identity transitions. To address this, we chose reprogramming to naive pluripotent stem cells (nPSCs) as a model system.

nPSCs have an unbiased potential to make all lineages of the developed organism, including the germ lineage. This fascinating identity arises naturally in the pre-implantation mammalian epiblast and can be captured *in vitro* as embryonic stem cells (hereafter referred to as nPSCs) or generated by reprogramming of differentiated cells back into induced nPSCs (inPSCs) (Takahashi and Yamanaka, 2006). Murine naive pluripotency can be maintained in culture by dual inhibition (2i) of Mek/Erk by PD03 and Gsk3 by Chiron, together with the Stat3 agonist LIF (Ying et al., 2008). Core members of the TF network regulating the naive identity include Oct4, Sox2, Nanog, Esrrb, Klf2, Klf4, Klf5, Stat3, and Tfcp2l1, and multiple inputs have been identified between the 2iLIF signal components and this network (reviewed in Martello and Smith, 2014).

In the post-implantation epiblast, the pluripotent cells have progressed to the primed state. This distinct identity exhibits markedly different transcriptional, epigenetic, and metabolic profiles and no longer gives rise to the germ lineage (reviewed in Morgani et al., 2017). These cells can be captured in culture as epiblast stem cells (EpiSCs) and require fibroblast growth factor (FGF) stimulation rather than inhibition of Mek/Erk signaling, together with the addition of ActivinA (FA) (Brons et al., 2007, Tesar et al., 2007).

Reprogramming of EpiSCs back to inPSCs provides several advantages as a model system to study cell identity transitions. The destination naive identity is extremely well defined in terms of its molecular signature, and functional assays such as clonogenic expansion and chimeric contribution leave no doubt as to whether the identity in question has indeed been generated. Reprogramming of EpiSCs requires only one driving naive factor combined with defined modulation of the signaling environment (Guo et al., 2009, van Oosten et al., 2012). This is in stark contrast to somatic cell reprogramming, which requires multiple genetic and signal variables to be introduced simultaneously to achieve reprogramming, prohibiting causal ascription of changes to individual inputs (reviewed in Smith et al., 2016). Furthermore, rapid naive gene expression responses follow transgene induction in EpiSCs, even while maintaining EpiSC FA culture conditions (Stuart et al., 2014). Thus, in this system, we can disentangle the contributions of TFs and signals to identity transition mechanisms and kinetics.

By use of individual, inducible factors coupled with independent manipulation of signal parameters, we interrogated how naive pluripotency is instructed by interplay between TFs and signals. We defined principles and mechanisms governing naive pluripotency establishment, which were also applicable to other contexts, including embryonic development and somatic cell reprogramming. Importantly, we provide explicit evidence of cellular identity as a multidimensional attractor state, with mechanistically as well as transcriptionally distinct pathways to transit between the same start and end identities.

## Results

### Reprogramming Initiation Is Driver Dependent

**Figure 1:**
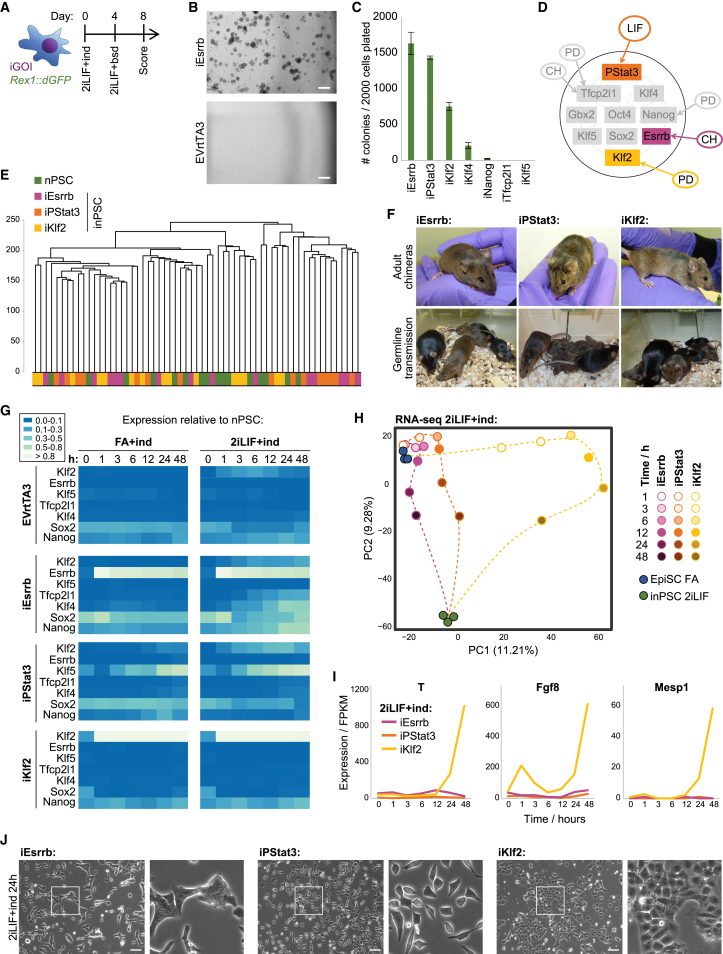
Reprogramming Initiation Is Driver Dependent (A) Reprogramming protocol for *Rex1*^*+/dGFP.IRES.bsd*^ (*Rex1::*dGFP) EpiSCs with individual, inducible driver genes of interest (iGOI). ind, induction of driver (GCSF for iPStat3, dox for others); bsd, blasticidin. (B) Phase images of iEsrrb and EmptyVector+rtTA3 (EVrtTA3, negative control) wells on day 8. Scale bars, 500 μm. (C) Mean number of inPSC colonies on day 8 ± SD (n = 3) per 2,000 cells plated. (D) Inputs of Esrrb, PStat3, and Klf2 to the naive network. Signals: PD, PD03; CH, Chiron. (E) Unsupervised hierarchical cluster of scRNA-seq, computed with the Ward.D2 agglomeration method and Euclidean distances for all expressed genes (fragments per kilobase per million [FPKM] > 0). (F) Blastocyst injection of inPSCs (agouti) yielded high-contribution adult chimeras capable of germline transmission (agouti pups). (G) Heatmap of mean gene expression from 0–48 h, measured by RT-qPCR relative to Gapdh and then normalized to nPSCs. (H) PCA based on variable genes (log_2_ FPKM > 1, CV^2^ > 0.5, calculated for each driver and then merged to a single list). (I) Expression of mesodermal markers following reprogramming induction in 2iLIF. (J) Phase images and indicated zooms 24 h after reprogramming induction. Scale bars, 100 μm. See also Figure S1.

To causally ascribe independent genetic and signal variables to reprogramming events, use of single drivers is necessary. We tested the reprogramming efficacy of individual naive factors in embryo-derived *Rex1*^*+/dGFP.IRES.bsd*^ (*Rex1::*dGFP) EpiSCs (Figures 1A–1C). Doxycycline (dox)-inducible (i) transgenes were used for Esrrb, Klf2, Klf4, Klf5, Nanog, and Tfcp2l1. Stat3 activation by phosphorylation (iPStat3) was elicited by GCSF stimulation of the GY118F receptor transgene (Burdon et al., 1999) because LIF signal transduction of EpiSCs is limited (Yang et al., 2010). iEsrrb, iPStat3, and iKlf2 were the most efficient single drivers in 2iLIF (Figure 1C). Interestingly, each inputs to the naive network along a different regulatory axis (Figure 1D): that of Chiron, LIF, and PD03 respectively (Martello et al., 2012, Niwa et al., 1998, Yeo et al., 2014). inPSCs established by these drivers were transcriptionally indistinguishable (Figures 1E and S1A) and were chimera and germline competent (Figure 1F), demonstrating molecular and functional equivalency. Therefore, we took iEsrrb, iPStat3, and iKlf2 as a model set of single reprogramming drivers for mechanistic study.

We analyzed the initial transcriptional response to each driver from 1–48 h (Figure 1G). In 2iLIF, naive gene upregulation by iPStat3 was moderate and by iEsrrb was substantially faster and stronger, whereas iKlf2 surprisingly did not upregulate naive genes and even silenced Sox2 (Figure 1G). These differing kinetics are further reflected by the rates of *Rex1::*dGFP upregulation and of transgene-independent inPSC formation from day 2 onward (Figures S1B and S1C) but are not attributable to differences in transgene induction kinetics or levels (Figures S1D and S1E).

To investigate the contribution of and interplay between genetic and signal variables, we compared driver induction in 2iLIF versus FA conditions from 1–48 h. For iPStat3 and iKlf2, responses were similar under both conditions (Figure 1G). However, for iEsrrb the response was highly condition dependent, with Esrrb and 2iLIF working in synergy to rapidly induce naive genes. Transcriptome-wide, iEsrrb and 2iLIF components interact to elicit a trajectory distinct from that of iEsrrb in FA (Figure S1F). In contrast, the signaling environment did not play a strong role in the early transcriptional behavior of iKlf2, with more similarity between time points than conditions (Figure S1F).

Considering that Klf2 is a potent reprogramming driver (Figure 1C), its initial lack of naive gene induction (Figure 1G) presented a fascinating conundrum. Principal-component analysis (PCA) showed a remarkable transcriptional divergence following Klf2 induction, corroborated by *k-*means cluster analysis (Figures 1H and S1G). We asked which genes could cause such a divergence and found robust upregulation of mesodermal markers in a Klf2-specific manner (Figure 1I). This indicates initial instigation of a different program downstream of Klf2 rather than simply a delayed naive induction kinetic.

Together, expression analyses revealed that the pattern and kinetics of naive network induction were driver dependent and that signal contribution was modulated by the driver (Figures 1G, 1H, S1F, and S1G). Morphological changes during reprogramming initiation were also driver specific (Figure 1J). Nonetheless, these divergent processes ultimately reconverged on the same naive pluripotent destination identity (Figures 1E, 1F, and S1A).

### Single-Cell RNA Sequencing (RNA-Seq) Defines Distinct Productive Trajectories

**Figure 2:**
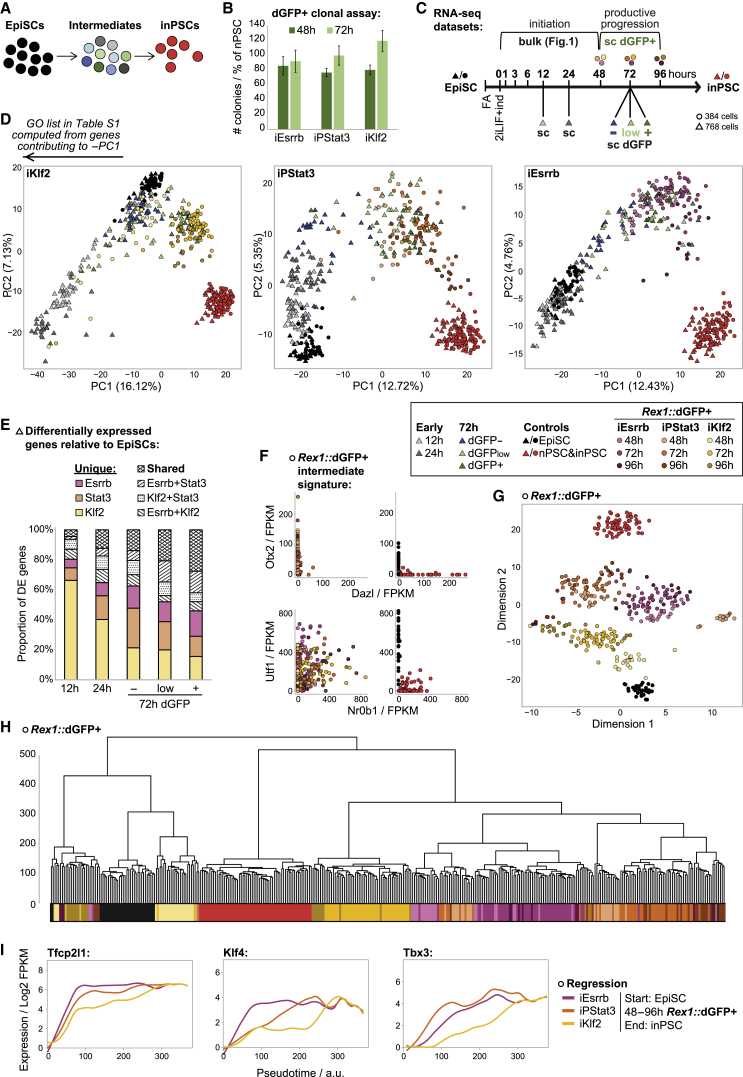
Single-Cell RNA-Seq Defines Distinct Productive Trajectories (A) Necessity to isolate productive intermediates for mechanistic study. (B) *Rex1::*dGFP+ cells were isolated by fluorescence-activated cell sorting (FACS) at 48/72 h and plated for clonal assay. Reprogramming intermediates were plated in 2iLIF+dox/GCSF and established *Rex1::*dGFP nPSCs in 2iLIF. Dox/GCSF was withdrawn and blasticidin was applied on day 6. Mean inPSC colonies ± SD (n = 3) scored on day 9 are indicated as percentage of nPSC colonies for each experiment. (C) Schematic summarizing RNA-seq datasets. (D) PCA based on variable genes (log_2_ FPKM > 1, CV^2^ > 0.5). (E) Numbers of unique and shared differentially expressed (DE) genes for each driver compared with EpiSCs. (F) Expression scatterplots of EpiSC markers (Otx2 and Utf1) and naive markers (Dazl and Nr0b1). (G) t-SNE plot showing relationships between single-cell transcriptomes. (H) Unsupervised hierarchical cluster computed with the Ward.D2 agglomeration method and Euclidean distances. (I) LOESS regression fit lines summarizing expression kinetics, computed from scatterplots of log_2_ FPKM versus pseudotime (Figures S2C and S2D) for single cells. See also Figure S2 and Video S1.

Because reprogramming to naive pluripotency is heterogeneous and asynchronous, cells undergoing the change of interest must be resolved from the average to study transition mechanisms (Figure 2A) (reviewed in Buganim et al., 2013). Therefore, we tested isolation of productively transitioning intermediates based on activation of the *Rex1*::dGFP reporter. *Rex1* is silent in EpiSCs, increases incrementally during reprogramming (Stuart et al., 2014), and is extensively characterized as a sensitive proxy of naive network strength (Kalkan et al., 2017). When replated in 2iLIF+dox/GCSF, we found that emergent destabilized GFP (dGFP)+ reprogramming intermediates were destined to form naive colonies with an efficiency comparable with nPSCs (Figure 2B).

We performed single-cell (sc) RNA-seq at 12 and 24 h (all cells), on dGFP–/low/+ at 72 h, and on dGFP+ at 48, 72, and 96 h (Figure 2C). With the former (triangles), we capture early differences and trajectory overviews, whereas the 48–96 h dGFP+ (circles) resolves cells undergoing productive progression to naive pluripotency. PCA revealed that, for iEsrrb and iPStat3, start EpiSCs and end inPSCs represent the extremes of identity along PC1. In contrast, iKlf2 shows a marked diversion in the first 12–24 h, away from both EpiSC and inPSC along PC1 (Figure 2D), corroborated by unsupervised hierarchical clustering (Figure S2A). To investigate the molecular features of this early diversion, we performed Gene Ontology (GO) analysis (Table S1). There was significant GO enrichment for processes involved in cell motility and development, consistent with initial diversion of iKlf2 cells in a mesodermal direction.

To further investigate trajectory distinctions, we performed differential gene expression analysis. We compared each sample with start EpiSCs to see how expression signatures changed over time for each driver and, by using a common reference, to assess similarities versus differences between drivers. We plotted Venn diagrams to find the numbers of differentially expressed (DE) genes that are unique to or shared between drivers at each time point (Figure S2B) and summarize these in Figure 2E. Drivers initially diverge, in particular with iKlf2 exhibiting 2,985 unique DE genes at 12 h. Over time, drivers then reconverge, indicated by the increasing proportion of shared DE genes. At 72 h, there is positive correlation between the dGFP level and the proportion of shared DE genes, consistent with approach of distinct trajectories to the common destination identity.

The initial divergence of iKlf2 cannot simply be attributed to an unproductive offshoot. iKlf2 72h dGFP– cells cluster back near the EpiSCs, not at the end of a different trajectory (Figure 2D). By live imaging, we confirmed that the divergent iKlf2 cells at 12/24 h are not undergoing cell death (Video S1). It logically follows that iKlf2 cells start on a divergent trajectory prior to acquisition of naive pluripotency.

Video S1. Initiation of iKlf2 EpiSC Reprogramming, Related to Figure 2

To connect early trajectory divergence with subsequent acquisition of naive pluripotency, we analyzed the 48–96 h dGFP+ cells in more detail. Intermediate identity was confirmed by naive versus EpiSC marker expression profiles (Figure 2F). Sample relationships assessed by t-distributed stochastic neighbor embedding (t-SNE) dimensionality reduction and hierarchical clustering revealed that dGFP+ sorted intermediates arranged by driver rather than time point (Figures 2G and 2H). This demonstrates that reprogramming routes are transcriptionally distinct throughout the productive transitions, not only during initiation. Again, the iKlf2 route was transcriptionally more different from those of iPStat3 and iEsrrb (Figures 1H, 2H, and S2A).

We examined the kinetics of naive network activation in single dGFP+ cells. To deconvolute the asynchronous nature of reprogramming, we ordered cells by fraction of similarity to origin EpiSCs and destination inPSCs to assign pseudotime coordinates (Figure S2C). iEsrrb exhibited the fastest kinetics of naive network induction for the majority of naive genes, whereas iKlf2 was slowest (Figures 2I and S2D). This is in agreement with the different kinetics observed in bulk analyses from 0–48 h (Figure 1G), now extended to 48–96 h and within dGFP+ single cells.

### iKlf2 Reprogramming Proceeds via a Mesoderm-like State

**Figure 3:**
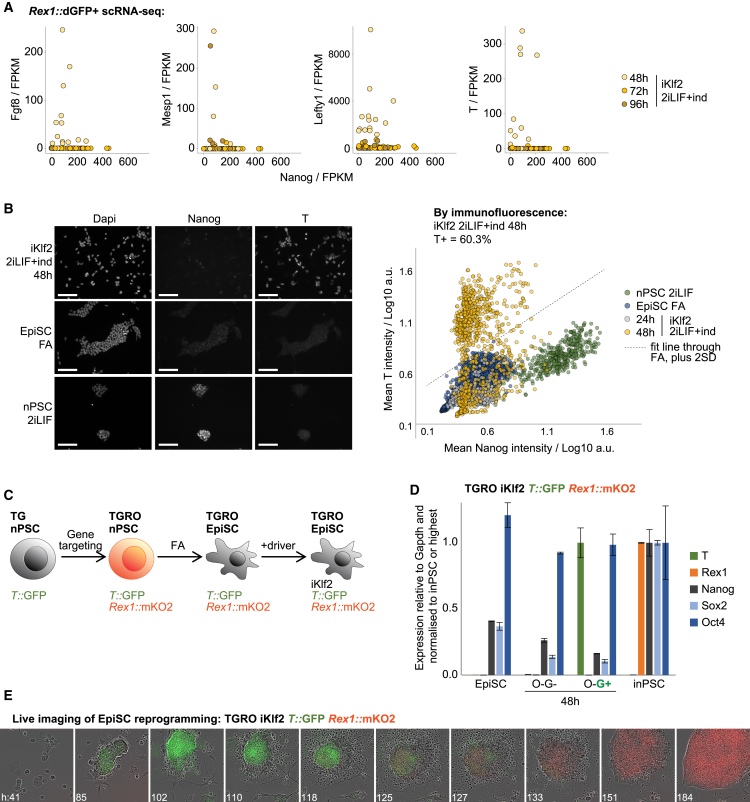
iKlf2 Reprogramming Proceeds via a Mesoderm-like State (A) Expression scatterplots of mesodermal markers versus Nanog. (B) Immunofluorescence for T and Nanog was quantified 24/48 h after iKlf2 dox induction (ind) of the original *Rex1::*dGFP EpiSCs on a total of 3,675 cells. To determine the percentage of T+ cells, a stringent threshold was calculated: mean of EpiSC values + 2 SD. Scale bars, 100 μm. (C) Strategy to generate *T/Rex1* double reporter (TGRO) iKlf2 EpiSCs. (D) RT-qPCR analyses following reprogramming induction of TGRO iKlf2 EpiSCs. *T::*GFP+ (G+) and *T::*GFP− (G−) populations were both *Rex1::*mKO2− (O−) at 48 h. Mean expression is displayed ± SD (n = 3). (E) Live imaging of TGRO iKlf2 EpiSC reprogramming. On day 4, iKlf2 induction was withdrawn, and blasticidin was added to select for inPSCs with active *Rex1* reporter. Merge snapshots are shown from Video S2. See also Figure S3 and Video S2.

For iKlf2, the upregulation of mesodermal markers observed during bulk initiation persisted in productive *Rex1::*dGFP+ single cells (Figures 1I, 3A, and S3A). This suggests that transient activation of mesodermal markers was not due to differentiation of a population of unproductive cells but was a transcriptional response occurring during productive establishment of naive pluripotency when driven by Klf2. T (Brachyury) is specifically expressed in and essential for nascent mesoderm formation. To determine the proportion of iKlf2 intermediates expressing T on the protein level, we performed and quantified immunofluorescence following iKlf2 induction (Figure 3B). By 48 h, we observed robust expression of T protein in 60% of iKlf2 cells, indicating that these are a major population.

To trace the outcome of these T+ intermediates through the reprogramming process, we generated *T/Rex1* double reporter EpiSCs (Figure 3C). Into *T::*GFP reporter nPSCs (*T*^*+/*GFP^; Fehling et al., 2003), we knocked monomeric Kusabira-Orange 2 fluorescent protein (mKO2) into the *Rex1* locus (Figure S3B). We obtained *T/Rex1* double reporter EpiSCs (TGRO) by differentiation for 10 passages in FA and then transfected iKlf2 reprogramming driver. We confirmed that these EpiSCs upregulate T in response to iKlf2 induction and verified that T and GFP expressions are in agreement (Figures 3D and S3C).

By live imaging, we traced the activity of *T* and *Rex1* during iKlf2-driven reprogramming of double reporter EpiSCs (Figure 3E; Video S2). T+ colonies emerge around day 2. Strikingly, these T+ colonies then convert into Rex1+ colonies around day 4. The largely sequential nature of *T* then *Rex1* reporter activation is consistent with the low percentage of T+ cells captured by scRNA-seq of Rex1+ intermediates (Figure 3A). Together, this provides direct evidence that productive iKlf2 reprogramming proceeds via a T+ state on the protein level, demonstrating diversion toward mesoderm prior to acquisition of naive pluripotency.

Video S2. Reprogramming of iKlf2 *T::*GFP *Rex1::*mKO2 EpiSCs, Related to Figure 3

### iPStat3 Reprogramming Proceeds via an Early ICM-like State

**Figure 4:**
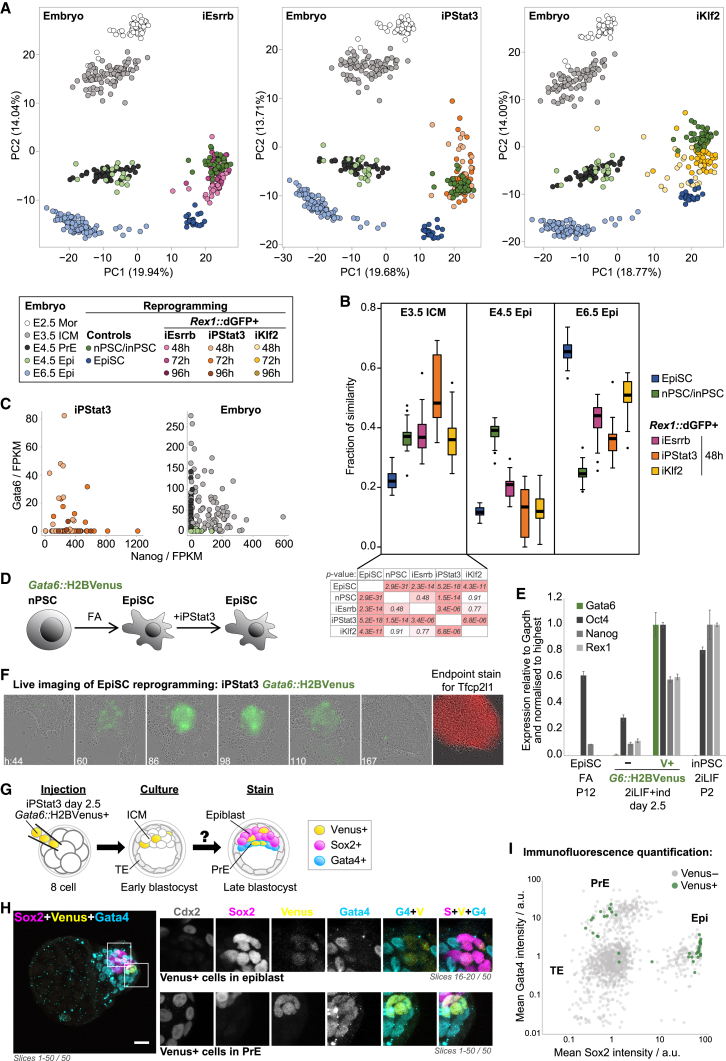
iPStat3 Reprogramming Proceeds via an Early ICM-like State (A) PCA based on variable genes (log_2_ FPKM > 1, CV^2^ > 0.5) for reprogramming intermediates and embryo single cells. Mor, compacted morula; ICM, inner cell mass; Epi, epiblast; PrE, primitive endoderm. PC1 separates *in vivo* versus *in vitro* datasets; PC2 portrays developmental progression. (B) Fraction of similarity to signature embryo datasets was computed by quadratic programming for each *in vitro* single cell and is presented as box-and-whisker plots. (C) Scatterplots of Gata6 versus Nanog for iPStat3 reprogramming and E3.5 and E4.5 embryos. (D) Strategy to generate *Gata6* reporter iPStat3 EpiSCs. (E) RT-qPCR analyses following GCSF induction (ind) of *Gata6::*H2BVenus iPStat3 EpiSCs. Mean expression is displayed ± SD (n = 3). (F) Live imaging of *Gata6::*H2BVenus iPStat3 EpiSC reprogramming. On day 4, iPStat3 induction was withdrawn. Merge snapshots are shown from Video S3. Endpoint staining identified inPSC colonies. (G) *Gata6*::H2BVenus+ iPStat3 day 2.5 reprogramming intermediates were injected into 8-cell embryos and then traced in resultant late blastocyst chimeras. (H) Maximum intensity Z-projections for a stained chimeric blastocyst. Scale bar, 20 μm. Zooms are shown of the indicated regions and slices for single channels and the indicated merges. Top: contribution of injected cells to Sox2+Gata4− epiblast is apparent. Although Gata6 is no longer expressed in the E4.5 epiblast, Venus has a long half-life, allowing us to trace contribution 2 days after injection of positive cells. Bottom: region with contribution of Venus+ cells to Sox2−Gata4+ PrE. Because Gata6 is still expressed in E4.5 PrE, contributing cells actively express Venus. (I) Quantification of Gata4 versus Sox2 staining in 7 embryos. See also Figure S4 and Video S3.

To place the reprogramming trajectories in the context of early development, we compared scRNA-seq of productive *Rex1::*dGFP+ intermediates with embryonic day 2.5 (E2.5)–E6.5 embryos (Deng et al., 2014, Mohammed et al., 2017). Single-cell transcriptome analyses revealed that iPStat3 reprogramming intermediates transiently acquired significant similarity to the early inner cell mass (ICM) (Figures 4A and 4B) and exhibited a Nanog+Gata6+ double-positive signature (Figures 4C and S4A). Nanog+Gata6+ co-expression is a hallmark of the early ICM (Plusa et al., 2008), prompting the hypothesis that iPStat3-driven reprogramming goes further back to an early ICM-like state and then forward into the consolidated naive identity. Indeed, the temporal sequence of naive gene activation in iPStat3 intermediates emulates that of the embryo (Figure S4B).

To functionally test the properties of Gata6+ iPStat3 reprogramming intermediates, we generated *Gata6* reporter EpiSCs by differentiation from *Gata6*^+/H2BVenus^ nPSCs (Freyer et al., 2015) and then transfected GY118F (iPStat3). We confirmed that resultant EpiSCs upregulate Gata6 in response to iPStat3 induction, that Gata6 and Venus expression are in agreement, and that Nanog+Gata6+ double-positive cells are present on the protein level (Figures 4D, 4E, and S4C). By live imaging of iPStat3-driven reprogramming, we observed Gata6+ cells emerge on days 2–3 (Figure 4F; Video S3). These subsequently gave rise to inPSCs by the endpoint, providing direct evidence that Gata6+ iPStat3 reprogramming intermediates are productive.

Video S3. Reprogramming of iPStat3 *Gata6::*H2BVenus EpiSCs, Related to Figure 4

The defining functional property of the early ICM is the potential to generate primitive endoderm (PrE, hypoblast) as well as the pluripotent epiblast. To test whether they acquire this greater potency, we injected Gata6+ iPStat3 reprogramming intermediates into 8-cell-stage embryos and then cultured to the late blastocyst stage, by which time the PrE and epiblast lineages are fully segregated. Chimeric embryos were fixed and analyzed for contribution of injected cells to the epiblast (Sox2+), PrE (Gata4+), and trophectoderm (Cdx2+) (Figure 4G). Remarkably, the Gata6+ population contributed to both epiblast and PrE, consistent with a gain of potency equivalent to that of the early ICM (Figures 4H and 4I). Gata6+ intermediates were Sox2+Gata4– prior to injection (Figure S4D), as the early ICM would be, and then could become either Sox2+Gata4– epiblast or Sox2–Gata4+ PrE in the embryo (Figures 4H and 4I). As expected, established inPSCs contributed only to epiblast, and EpiSCs did not contribute at all (data not shown).

In sum, the iPStat3 reprogramming population transiently gains resemblance to the early ICM, both in terms of its molecular signature and its developmental potency.

### Routes Have Distinct Genetic and Signal Requirements

**Figure 5:**
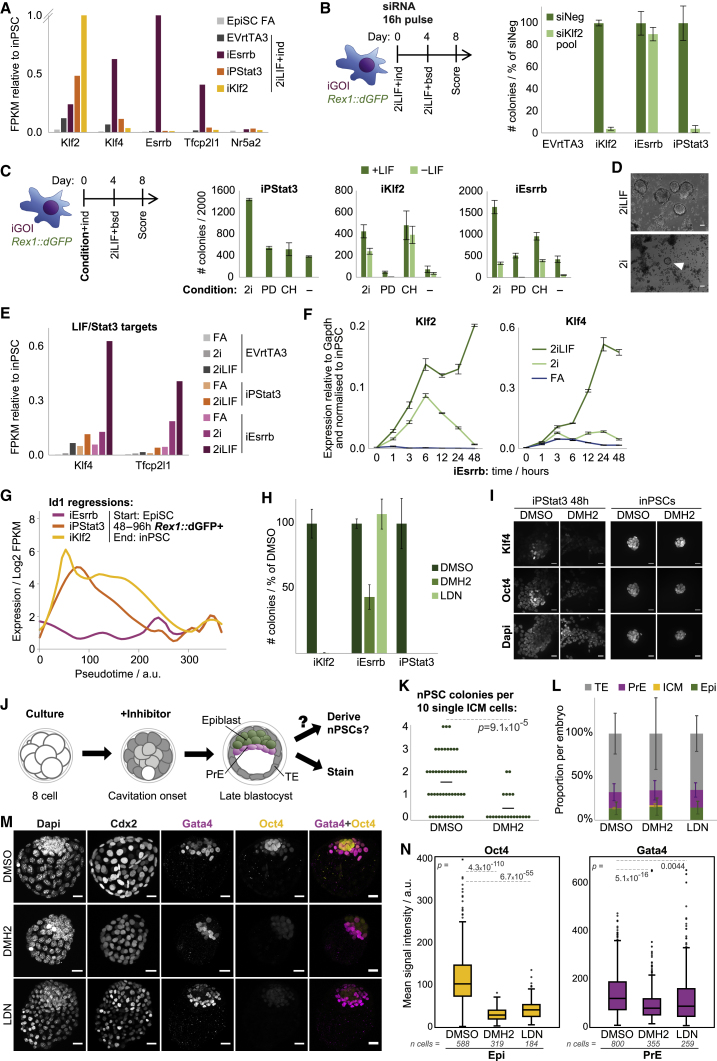
Routes Have Distinct Genetic and Signal Requirements (A) Gene expression after 24 h relative to inPSCs. y-axis: iEsrrb, Esrrb = 3.32; iKlf2, Klf2 = 8.30. (B) KD was performed at reprogramming onset with a single pulse of small interfering (si) RNA. Mean inPSC colonies scored on day 8 are presented ± SD (n = 3). (C) Reprogramming was induced under different conditions from days 0–4 and then selected in 2iLIF+blasticidin. inPSC colonies scored on day 8 are presented as mean ± SD (n = 3). 2i, PD+CH. (D) Phase images of iEsrrb on day 8 in 2iLIF+blasticidin after reprogramming from days 0–4 in 2iLIF+dox or 2i+dox as indicated. The arrowhead indicates an inPSC colony. (E) Expression of LIF/Stat3 target genes 24 h after driver induction under the indicated conditions. (F) Timecourse RT-qPCR analyses of iEsrrb EpiSCs under the indicated conditions + dox. Mean expression is displayed ± SD (n = 3). (G) LOESS regression fit lines summarize Id1 kinetics during reprogramming, computed from log_2_ FPKM versus pseudotime for single cells (Figure S2C). (H) 3 μM DMH2, 0.6 μM LDN, or DMSO were applied to reprogramming in 2iLIF+dox/GCSF from days 0–4, and then inPSCs were selected in 2iLIF+blasticidin. inPSC colonies scored on day 8 are presented as mean ± SD (n = 3). (I) Immunofluorescent staining after 48 h of inhibitor treatment for iPStat3 reprogramming in 2iLIF+GCSF or for previously established iPStat3 inPSCs in 2iLIF. Scale bars, 20 μm. (J) Schematic summarizing BMP inhibitor treatment of pre-implantation embryos. (K) Quantitative nPSC derivation following embryo treatment with DMSO or 3 μM DMH2. nPSC colonies were scored per 10 single ICM cells. Black line, mean. DMSO, n = 7; DMH2, n = 8 embryos. (L–N) Late blastocysts were stained for Cdx2, Gata4, and Oct4 following treatment with DMSO, 3 μM DMH2, or 0.3 μM LDN. (L) Mean cell number per lineage ± SD, presented as a proportion of the total cells per embryo. DMSO, n = 23; DMH2, n = 18; LDN, n = 7 embryos. (M) Representative maximum intensity Z-projections and indicated merge. Scale bars, 20 μm. (N) Quantification of immunofluorescent signal for Oct4 in Epi nuclei and Gata4 in PrE nuclei, presented as box-and-whisker plots. DMSO, n = 23; DMH2, n = 18; LDN, n = 7 embryos. See also Figure S5.

To test whether the divergent transcriptional trajectories are indicative of mechanistic differences, we assessed their genetic and signal requirements. Putative downstream genetic mediators were identified by examining the expression of known reprogramming drivers 24 h after induction of iEsrrb, iKlf2, or iPStat3 (Figures 1G, 5A, and S5D). Endogenous Esrrb was not upregulated by either iKlf2 or iPStat3 by 24 h, and correspondingly its knockdown (KD) did not prevent reprogramming (Figures S5A and S5B). In contrast, endogenous Klf2 reached 50% and 20% of nPSC levels in iPStat3 and iEsrrb, respectively. Given this early response, we tested whether Klf2 is a mediator of iPStat3- or iEsrrb-driven reprogramming. Transient Klf2 KD abolished reprogramming driven by iPStat3 but not iEsrrb (Figures 5B, S5A, and S5B). This implicates Klf2 as a critical mediator of reprogramming initiation by iPStat3. Klf2 is not considered a PStat3 target in nPSCs, implying different network topologies during establishment versus maintenance of naive pluripotency. Curiously, iPStat3 sensitivity to Klf2 KD was context dependent and partially alleviated in the absence of PD03 (Figure S5C).

To assess route differences in terms of exogenous signal requirements, we challenged the first 4 days of reprogramming with 2iLIF signal permutations (Figure 5C). iPStat3 yielded inPSCs in the absence of both PD03 and Chiron, but together, PD03 and Chiron synergistically boosted the efficiency. However, the effect of PD03 and Chiron was driver dependent: Chiron was essential for iKlf2-driven reprogramming, with no benefit from additional supplementation with PD03. Functional redundancy between Klf2 and PD03 has been noted previously (Yeo et al., 2014), and the inability of iKlf2 to drive reprogramming without direction from an exogenous signal is in agreement with the observation that iKlf2 does not directly induce naive gene expression (Figure 1G). Unlike iKlf2, reprogramming driven by iEsrrb was highly LIF dependent (Figures 5C and 5D). iEsrrb induction in LIF led to greater upregulation of canonical PStat3 targets than induction of iPStat3 itself (Figure 5E). This was not due to elevation of PStat3 protein by Esrrb (Figure S5D) and thus demonstrates downstream synergy between Esrrb and PStat3. To identify when this synergy became effective, we performed timecourse expression analyses. A turning point occurred 6 h after Esrrb induction. From 0–6 h, Klf2 was upregulated similarly in 2i with or without LIF for both iEsrrb and negative control EpiSCs; after 6 h, Klf2 expression continued to increase in iEsrrb+2iLIF but collapsed in iEsrrb+2i and all control conditions (Figure 5F and S5E). Klf4 upregulation also launched in earnest after 6 h with iEsrrb+2iLIF.

In light of the above observations that signal requirement and interpretation are driver dependent, we interrogated *Rex1::*dGFP+ 48–96 h scRNA-seq data for evidence of other signaling differences between iKlf2, iEsrrb, and iPStat3 productive intermediates. BMP signaling pathway target Id1 is upregulated in iKlf2 and iPStat3, but not iEsrrb (Figure 5G). Id1 upregulation is intermediate-specific, with negligible expression in starting EpiSCs or destination inPSCs. BMP signaling is a key pluripotency-sustaining component in the serum of classical nPSC cultures (Ying et al., 2003), is important for mesenchymal–epithelial transition (MET) in serum-based fibroblast reprogramming (Samavarchi-Tehrani et al., 2010), but is not active in 2iLIF-cultured nPSCs (Boroviak et al., 2014). We assessed BMP pathway status by PSmad1/5 immunofluorescence during EpiSC reprogramming in 2iLIF, finding positive staining for iKlf2 and iPStat3 but not iEsrrb (Figure S5F). Therefore, BMP signaling is activated in a route-specific manner.

To test whether auto/paracrine BMP signaling is required during EpiSC reprogramming, we applied BMP inhibitor from days 0–4. DMH2 is a specific and well-characterized BMP receptor inhibitor (Figure S5G; Hao et al., 2010), and we also verified key findings with a different inhibitor, LDN193189 (LDN) (Cuny et al., 2008). BMP inhibition abolished iKlf2- and iPStat3-driven reprogramming in 2iLIF, but inPSC colonies still formed for iEsrrb (Figure 5H). Therefore, BMP inhibition blocked reprogramming only in lines exhibiting evidence of active BMP signaling in their intermediates. This was specific to the transition, being dispensable for maintenance of the resultant inPSCs in 2iLIF (Figure 5I; S5H).

Together, these results demonstrate that iKlf2, iPStat3, and iEsrrb drive reprogramming by mechanistically distinct routes in terms of their genetic and signal requirements and their differential modulation of exogenous and endogenous signal transduction.

### BMP Signaling Is Required for Naive Pluripotency Establishment *In Vivo*

Having identified BMP signaling requirements in two routes of reprogramming, and given that iPStat3 reprogramming intermediates transiently acquired similarity to the early ICM, we explored whether endogenous BMP signaling also plays a role in naive pluripotency establishment *in vivo*. The BMP signaling pathway is active in pre-implantation mouse embryos from the 4-cell stage onward, including in the ICM (Graham et al., 2014, Reyes de Mochel et al., 2015), so involvement in epiblast specification is plausible. We applied BMP inhibitor to the late morula, cultured embryos to the late blastocyst stage, then analyzed the effect on each lineage and performed quantitative nPSC derivation (Figure 5J). Per cell, we observed a 4-fold reduction in nPSC derivation efficiency from embryos that had been treated previously with BMP inhibitor (p = 9.1 × 10^−5^), demonstrating that BMP inhibition had disrupted pluripotency establishment in the embryo (Figures 5K and S5I).

By analysis of immunofluorescence, we counted the number of cells in Epi, PrE, and trophectoderm (TE) lineages, and quantified the intensity of lineage marker expression (Figures 5L–5N and S5J–S5L). The proportions of cells assigned to each lineage were unaffected by BMP inhibition (Figure 5L). PrE and TE exhibited either mildly reduced (DMH2) or unaffected (LDN) lineage marker expression, whereas Oct4 expression in the Epi lineage was dramatically reduced by both inhibitors (p = 4.3 × 10^−110^ for DMH2; p = 6.7 × 10^−55^ for LDN) (Figures 5M, 5N, and S5K). We also performed Nanog staining on a subset of embryos and observed a significant reduction in the Epi lineage for both inhibitors (Figure S5L).

In sum, we found that BMP inhibition had a specific effect on naive pluripotency establishment in the embryo, dramatically reducing Epi marker expression and the functional ability to yield nPSCs despite a normal proportion of cells being allocated to the Epi compartment. Identification of this role for the BMP pathway *in vivo* highlights the power of our defined reprogramming systems to uncover principles of identity specification.

### A Defined Oct4 Level Is a Common Feature of All Routes

**Figure 6:**
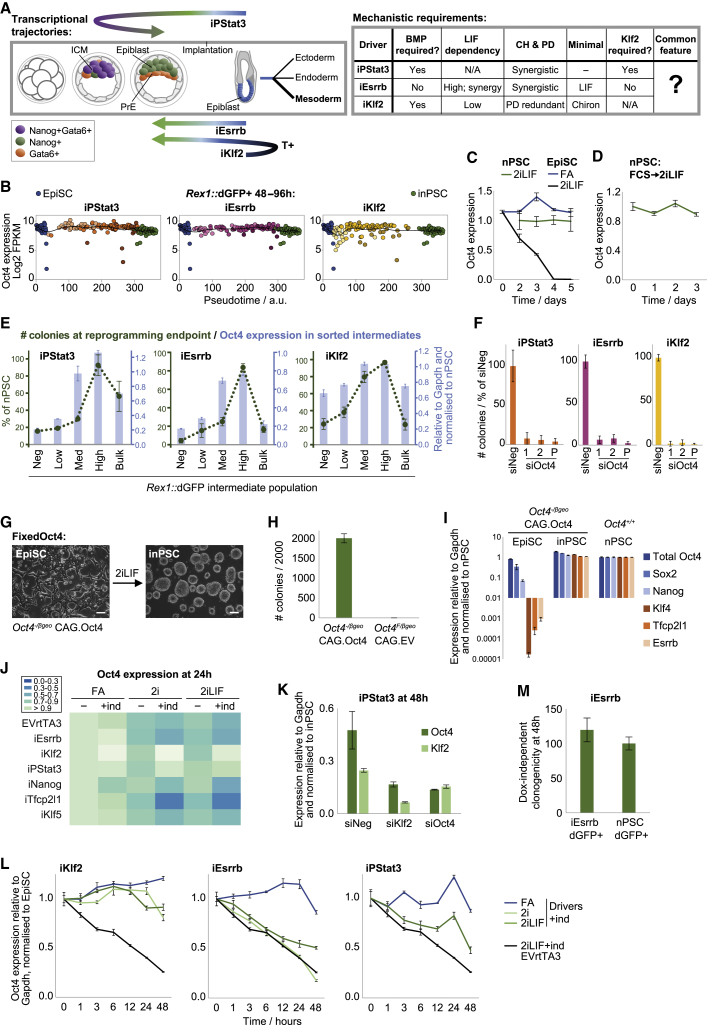
EpiSC Reprogramming Converges on the Fine-Tuning of Oct4 Expression (A) Summary of transcriptional trajectories and mechanistic requirements for each driver. (B) Scatterplots of Oct4 expression in single cells versus pseudotime (Figure S2C), fitted with LOESS regression lines. (C and D) Timecourse RT-qPCR analyses of mean Oct4 expression, displayed relative to Gapdh and normalized to nPSCs ± SD (n = 3). (C) EVrtTA3 control EpiSCs in FA or 2iLIF+dox and nPSCs maintained in 2iLIF. (D) nPSCs previously cultured in FCS+LIF and then switched to 2iLIF. (E) *Rex1::*dGFP negative, low, medium, high, and bulk reprogramming intermediates were isolated by flow cytometry, analyzed for average Oct4 expression level by RT-qPCR (blue), and then replated for clonogenicity assay in 2iLIF (green). Means are presented ± SD (n = 3). (F) Oct4 KD was performed at reprogramming onset with a single pulse of siRNA. inPSC colonies scored on day 8 are presented as mean ± SD (n = 3). P, pool. (G–I) FixedOct4 EpiSCs formed inPSC colonies at high efficiency in 2iLIF, indicated morphologically (scale bars, 100 μm) (G), by mean inPSC colonies scored on day 8 ± SD (n = 3) (H), and by RT-qPCR analyses (I). (J) Heatmap of Oct4 expression after 24 h, measured by RT-qPCR relative to Gapdh and then normalized to EpiSCs. (K) Oct4 or Klf2 KD was performed at iPStat3 reprogramming onset. After 48 h, expression was analyzed by RT-qPCR. (L) Timecourse RT-qPCR analyses for EpiSCs under the indicated conditions. Mean Oct4 expression is displayed ± SD (n = 3). EVrtTA3 control is shared between plots. (M) *Rex1::*dGFP+ iEsrrb reprogramming intermediates (2iLIF+dox) and nPSCs (2iLIF) were isolated by FACS at 48 h and plated for clonal assay in 2iLIF without dox. Blasticidin was applied on day 6. Mean naive colony number scored on day 9 is presented as percentage of nPSC colonies ± SD (n = 3). See also Figure S6.

The aforementioned differences in transcriptional trajectories, signal, and genetic requirements demonstrate that iKlf2, iPStat3, and iEsrrb instruct reprogramming by distinct mechanisms (Figure 6A). Given that the starting and destination cellular identities are the same in all three cases (Figures 1E and 1F), the extent of the route differences was surprising. Therefore, we asked whether there was a common feature that could reconcile the disparate transition logics.

From 48–96 h in *Rex1::*dGFP+ single cells, we found that Oct4 is expressed at endogenous pluripotent level, irrespective of the driver (Figure 6B). Maintenance of Oct4 throughout the transitions is not to be taken for granted. Although Oct4 is expressed at similar levels in EpiSCs and inPSCs (Figure S6A), this expression is supported by different transcriptional networks and driven from different enhancer elements (Tesar et al., 2007, Yeom et al., 1996). Indeed, signal switch of control EpiSCs from FA to 2iLIF triggered Oct4 downregulation (Figure 6C). In contrast, Oct4 was unperturbed in nPSCs upon switching from serum+LIF to 2iLIF (Figure 6D), indicating that 2i itself did not suppress Oct4 in a context where cellular identity was constant. Timecourse RT-qPCR analyses showed that Oct4 was expressed at or above PSC level in the dGFP+ reprogramming subpopulation from 48 h onward, but not always in the dGFP– subpopulation (Figure S6B). Together, this suggests that signal-mediated collapse of the primed network prior to naive network construction leads to Oct4 expression loss, creating a “vulnerable window” between different self-renewing Oct4-supporting configurations. Because 2iLIF triggered Oct4 collapse in control EpiSCs (Figure 6C), we reason that the observed maintenance of Oct4 in 2iLIF during productive reprogramming is an active process coordinated by the driving transgene (Figure 6B).

To evaluate the relationship between Oct4 level and productive reprogramming, we subdivided intermediate populations based on a finer gradient of *Rex1::*dGFP, measured Oct4 expression, and replated for clonogenicity assay in 2iLIF. Average Oct4 expression positively correlated with the subsequent reprogramming efficiency of a given subpopulation (Figures 6E and S6C). To test whether Oct4 maintenance is required for reprogramming, we performed transient Oct4 KD by a single pulse of siRNA treatment at reprogramming onset. inPSC formation was abolished (Figures 6F and S6D).

### Fixed Oct4 Expression Is Sufficient for Naive Instruction under Minimal Conditions

Having demonstrated that Oct4 maintenance is observed in and required for productive reprogramming, next we asked whether Oct4 maintenance is sufficient. We generated *Oct4*-null EpiSCs that constitutively express ectopic Oct4 at endogenous PSC level (FixedOct4) (Figures 6G–6I and S6E), according to methodology described by Radzisheuskaya et al. (2013). This uncouples Oct4 expression from identity or environmental perturbations; i.e., it prevents the loss of Oct4 upon switching of EpiSCs to 2iLIF. An *Oct4*-null background was necessary to ensure maintenance of total Oct4 levels and to avoid overexpression of Oct4, which triggers differentiation (Niwa et al., 2000). Correspondingly, ectopic Oct4 expression on top of a wild-type background gives very inefficient EpiSC reprogramming (Guo and Smith, 2010, Yang et al., 2019).

Following medium switch to 2iLIF, FixedOct4 EpiSCs rapidly generated inPSC colonies with extremely high efficiency (Figures 6H and 6I). The naive network response to FixedOct4 reprogramming initiation in 2iLIF has aspects in common with each of the other drivers but is overall most similar to iPStat3 (Figures 1G and S6F). We tested the signal dependencies of FixedOct4 reprogramming and found that LIF was the minimal requirement for naive pluripotency induction (Figures S6G and S6H). In FixedOct4 reprogramming, impetus toward the naive identity is provided only by exogenous signals; Oct4 is expressed equally in both EpiSCs and inPSCs, so there is no naive-specific transgene. Therefore, maintenance of Oct4 permits the identity transition, whereas signals such as LIF specify the direction.

### Reconciliation of Route Differences with Common Oct4 Maintenance

Despite distinctions between routes in terms of their transcriptional trajectories and mechanistic requirements (Figure 6A), Oct4 maintenance is a common feature that is required and sufficient for reprogramming (Figures 6F–6I). Now we reconcile route-specific attributes with this common denominator.

First we assessed the ability of each driver to rescue the drop in Oct4 expression when EpiSCs are treated with 2iLIF for 24 h (Figure 6J). Klf2 induction yielded the most effective Oct4 rescue, including on the protein level (Figures S6I–S6K). This Oct4 support could explain the high efficiency of Klf2-driven reprogramming despite its paradoxical dearth of naive gene induction (Figure 1G). iPStat3 also maintained Oct4 expression (Figure 6J). However, the remaining drivers failed to rescue the Oct4 drop in bulk populations.

Because Klf2 is the most effective supporter of Oct4 (Figure 6J) and is an early transcriptional responder to iPStat3 (Figure 5A), we asked whether these observations can be conceptually integrated. Transient Klf2 KD at iPStat3 reprogramming onset resulted in a 65% reduction of Oct4 expression (Figure 6K) and abolished iPStat3-driven reprogramming (Figure 5B). In contrast, Klf2 KD did not abolish reprogramming of FixedOct4 EpiSCs (Figure S6L) even though this is a highly LIF/Stat3-dependent process (Figures S6G and S6H). This places Oct4 maintenance as a functionally important downstream mechanism of Klf2 in reprogramming, likely to be direct because of its manifestation within 1 h (Figure 6L).

iEsrrb was the most efficient of all tested drivers (Figure 1C) but exhibited an initial drop in Oct4 expression at 24 h (Figures 6J, S6J, and S6K) prior to recovery in the productive subpopulation by 48 h (Figures 6B, S6B, and S6K). The outstanding feature of iEsrrb reprogramming initiation was rapid and strong upregulation of naive genes in a highly 2iLIF-dependent manner (Figures 1G, 5E, and 5F). To test whether this corresponded to rapid wiring of a coherent self-renewing naive network, we challenged the transgene-independent clonogenicity of iEsrrb *Rex1*::dGFP+ cells at 48 h by replating single sorted cells in 2iLIF without dox. Strikingly, their dox-independent clonogenicity was comparable with nPSCs (Figure 6M), indicating that, 48 h post-induction, a functional naive network has already formed for iEsrrb. Thus, we propose that iEsrrb drives a rapid transition between primed and naive networks, rescuing Oct4 expression within the vulnerable window between different self-renewing states.

Together, these results indicate that, irrespective of the mechanism used by different routes, achieving a PSC level of Oct4 is the common feature of successful reprogramming. This event creates the opportunity for transition into naive pluripotency, which is effected provided there is a conducive signal environment.

### A PSC Level of Oct4 Is Sufficient for Somatic Cell Reprogramming

**Figure 7:**
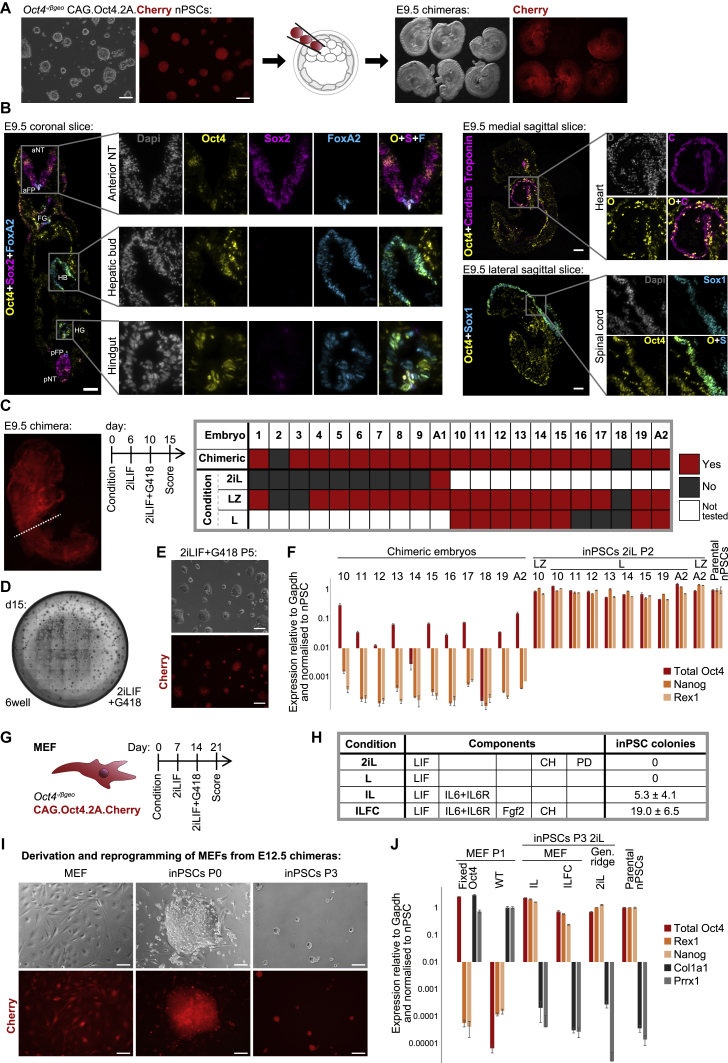
A PSC Level of Oct4 Is Sufficient for Somatic Cell Reprogramming (A) FixedOct4 nPSCs were injected into E3.5 C57BL/6 blastocysts and then transferred to recipients. The resultant embryos were collected at E9.5. Phase and Cherry images are shown of 5 chimeras and 1 negative control from the same litter. (B) The contribution of FixedOct4 cells to E9.5 chimeras was assessed by immunostaining of 8 μm cryosections. Zooms are shown of the indicated regions for single channels and indicated merges. Scale bars, 100 μm. NT, neural tube; FP, floor plate; FG, foregut; HB, hepatic bud; HG, hindgut; a, anterior; p, posterior. (C–J) Reprogramming of FixedOct4 cells from E9.5 chimeras and E12.5 MEFs. (C) The anterior portion of each E9.5 chimera was dissociated manually, subdivided into quarters, and then cultured under the indicated conditions in duplicates. Generation of inPSCs is summarized in the table. L, LIF; Z, aza; A, allantois. (D) inPSCs at P0 following reprogramming of one-eighth of an E9.5 chimera in one 6-well. (E) inPSCs at P5. Scale bars, 100 μm. (F) RT-qPCR analyses of inPSCs at P2 after reprogramming from E9.5 in L or LIFaza (LZ), followed by 2iLIF. Mean expression is shown ± SD (2 technical replicates per embryo). (G) Reprogramming protocol for FixedOct4 MEFs after derivation from E12.5 chimeras. (H) Conditions tested during the first week of MEF reprogramming. The number of inPSC colonies scored at day 21 is shown as mean ± SD (n = 3) per 5,000 MEFs plated. (I) FixedOct4 MEFs, an inPSC colony on day 21, and P3 inPSCs. Scale bars, 100 μm. (J) RT-qPCR analyses of FixedOct4 and wild-type MEFs, and FixedOct4 MEF-derived inPSCs after reprogramming in IL or ILFC followed by 2iLIF, and after derivation directly in 2iLIF for the genital ridge. Mean expression is shown ± SD (n = 3). See also Figure S7.

To address the applicability of our findings to other contexts, we derived somatic cells from FixedOct4 nPSCs by differentiation in chimeras (Figure 7A). Extensive analysis of E9.5 chimera cryosections confirmed *bona fide* development with widespread contribution of FixedOct4 cells to all germ lineages, expressing appropriate tissue-specific markers together with Oct4 (Figures 7B and S7A–S7D). FixedOct4 nPSCs were also capable of performing tetraploid complementation, a stringent assay for developmental contribution (Figure S7E).

Having verified contribution of FixedOct4 cells to downstream lineages in E9.5 chimeras, we tested whether they could reprogram and whether signal instruction was sufficient. After discarding a generous tail portion to stringently avoid germ cell contamination, we dissociated and cultured the anterior portion of each chimera to test reprogramming ability using three different conditions (Figure 7C): directly in 2iLIF, in LIF only, or in LIF combined with a low dose of 5-azacytidine (aza, an inhibitor of DNA methyltransferase activity, in case assistance was required to remodel a more constrained epigenetic landscape). After 6 days, all conditions were swapped to 2iLIF (Figure 7C). With the exception of positive control allantois, inPSCs were not generated when plated directly in 2iLIF, consistent with our previous demonstration that, when applied from the beginning, 2iLIF does not support somatic cell reprogramming (Silva et al., 2008). However, inPSCs were generated from 16 of 17 chimeras following culture in LIF+aza and from 7 of 9 chimeras after LIF only (Figures 7C–7F and S7F). Therefore, LIF is sufficient to induce reprogramming of FixedOct4 cells from E9.5 states as well as from EpiSCs (Figures S6G and S6H).

To test more developmentally advanced starting material, we derived FixedOct4 fibroblasts from E12.5 chimeras and investigated whether they could reprogram under signal instruction alone (Figures 7G–7J). E12.5 is a standard stage for murine embryonic fibroblast (MEF) derivation as the starting material for somatic cell reprogramming. FixedOct4 MEFs exhibited normal morphology (Figure 7I) and expressed both Oct4 and MEF markers (Figure 7J). Because MEF reprogramming usually takes longer than from EpiSCs and has different signal requirements in the early stages, we tried various conditions in the first week (Figures 7G and 7H). On day 7, we swapped all conditions to 2iLIF, and then, on day 14, applied G418 to select for inPSC colonies. As expected, direct application of 2iLIF did not yield inPSCs from MEFs but did allow derivation of naive pluripotent colonies from genital ridges (positive control). Unlike from FixedOct4 EpiSCs and E9.5 cells, LIF alone was insufficient to reprogram MEFs. Because MEFs may not effectively transduce the LIF signal, we added interleukin-6 (IL6) and soluble IL6 receptor (IL6R) to assist with Jak/Stat pathway activation. We also tested addition of FGF2 and Chiron because there is precedent for a positive role of these signals in fibroblast reprogramming (Giulitti et al., 2019, Li et al., 2011). We obtained inPSCs from IL6+IL6R+LIF+FGF2+Chiron (ILFC) and IL6+IL6R+LIF (IL) (Figures 7H–7J). Although ILFC was more efficient, IL represents the minimum requirement for MEF reprogramming.

This defines fine-tuned Oct4 expression together with Jak/Stat signaling as sufficient for naive pluripotency induction from a range of cell types: EpiSCs, E9.5 cells, and E12.5 MEFs.

## Discussion

We show that there are multiple routes by which naive pluripotency can be established from EpiSCs, with the unifying feature of active Oct4 maintenance. Not only do these routes differ in their transcriptional trajectories but, crucially, also in their mechanistic attributes of genetic and signal requirements (Figure 6A). Nevertheless, the molecular and functional equivalency of resultant inPSCs demonstrates that these routes ultimately converge to a single identity (Figures 1E and 1F). Thus, there is considerable flexibility for the specification of a single identity from a single origin. This adds further complexity to the paradigm of multicellular biology by which TFs and signals are used in different permutations and contexts to generate different cell types: they can also be used in different ways to generate the same cell type.

We relate reprogramming routes to development by transcriptome comparison, reporter live imaging, and *in vivo* lineage tracing. iPStat3 intermediates transcriptionally resemble the early embryo ICM and, remarkably, gain its greater developmental potency (Figure 4). In contrast, the iKlf2 route acquires a mesodermal signature prior to naive pluripotency induction (Figure 3). Therefore, initially moving backward or forward in developmental time can be compatible with successful reprogramming, provided key mechanistic criteria are met (Figure 6).

Adachi et al. (2018) recently reported that Esrrb acts as a pioneer TF during EpiSC reprogramming, binding to closed chromatin and recruiting P300 transcriptional coactivator in a LIF/Stat3-dependent manner. This is consistent with our observation that Esrrb-driven reprogramming is highly LIF-dependent (Figures 5C–5F). Stat3 and Smad1 are reported to form a protein complex together with P300 under conducive signaling conditions (Onishi et al., 2014), compatible with our finding that iPStat3-driven reprogramming is blocked by BMP signaling inhibition (Figure 5H). Based on this, we speculate that different reprogramming drivers engage with P300 via different partners, and that this might underpin their different mechanistic requirements (Figure 6A).

iKlf2 is enigmatic as an efficient EpiSC reprogramming driver. Its dearth of naive gene upregulation within the first 48 h is counterintuitive, as is its highly divergent initiation trajectory (Figures 1, 2, and 3). In the first 48 h, the only positive effect of iKlf2 on pluripotency genes is robust support of Oct4 expression (Figure 6). Because FixedOct4 is sufficient for highly efficient reprogramming, we reason that a similar phenomenon happens here: iKlf2 intermediates are Oct4+ and, thus, remain permissive for reprogramming directed by signals. We note that Oct4 is initially maintained during mesendoderm lineage entry (Downs, 2008, Thomson et al., 2011) and reason that transient lineage diversion can benefit reprogramming when it helps to achieve the Oct4 maintenance requirement. This signifies a conceptual shift, exposing expression of a “transition factor” as more important than the transcriptional program directly induced by a driver. Therefore, identity change does not simply require activation of the destination program but, instead, pivots on the mechanism that permits a transition to occur.

Ultimately, successful reprogramming routes can be thought of as different strategies that converge on the unifying, required, and sufficient feature of fine-tuned Oct4 expression (Figure 6). In light of this, we propose the following hypothesis: for a given EpiSC reprogramming driver, there is a certain probability of rescuing Oct4 during the vulnerable window after Oct4 loses support from the collapsing primed network. We suggest that reprogramming efficiency correlates with this probability, which is determined by (1) the ability of that factor itself to drive Oct4 expression and (2) the speed at which that factor orchestrates a coherent naive network to support Oct4 in an alternative topology. iKlf2 and iEsrrb occupy opposite extremes within this model, relying solely on the former and latter strategies, respectively (Figure S7G).

Results from other contexts further demonstrate that identity transition into naive pluripotency pivots on precise Oct4 expression. A PSC level of Oct4 is the minimal requirement for naive pluripotency induction not only from EpiSCs but also from developmentally more advanced cell types, including MEFs (Figure 7). In agreement with this, Liu et al. (2018) recently reported that CRISPR-based chromatin remodeling of the *Oct4* locus is sufficient to reprogram MEFs, using the acetyltransferase domain of P300 to activate endogenous Oct4. Thus, precise Oct4 expression is the defining feature in distinct contexts of nuclear reprogramming. It will now be interesting to explore how our findings relate to other advances made toward the optimization and understanding of induced pluripotency.

Although Oct4 expression at PSC level is required and sufficient for reprogramming under signal instruction, it is also compatible with *bona fide* development when returned to the embryo. In our FixedOct4 system, opposing but highly efficient identity transitions occur depending solely on the environment: induction of naive pluripotency in the presence of LIF (Figure 6) or re-entry to development *in vivo* (Figure 7). Oct4 plays a transition-permitting role during early differentiation of several lineages (Niwa et al., 2000, Radzisheuskaya et al., 2013) and can be briefly utilized to promote direct transdifferentiation from a fibroblast to a neural identity (Thier et al., 2012). In this light, and considering that low-Oct4 traps nPSCs in self-renewal (Karwacki-Neisius et al., 2013, Radzisheuskaya et al., 2013), we now define Oct4 as a “transition factor” permitting identity change in various directions depending on the context.

Our work supports theories that cell identities are multidimensional attractors, occupying local minima of stable network states (Huang et al., 2005, Kauffman, 1993). Here we provide a substantial advance on previous works, reaching a single destination identity via three different trajectories. Mechanistic as well as transcriptional differences verify that transitions occur via truly distinct intermediate states. Furthermore, we reveal the logic underpinning multidimensional access to the single attractor: fine-tuned support of a transition factor; in this case, Oct4. This provides a conceptual framework for the understanding of cell identity transitions. In the future, it will be of interest to continue identifying the transition factors and supporting logic for the multitude of developmental, regenerative, and pathological cell identity transitions.

## STAR★Methods

### Key Resources Table

**Table:** 

REAGENT or RESOURCE	SOURCE	IDENTIFIER
**Antibodies**		

Monoclonal mouse anti-Cardiac Troponin	Abcam	Cat#ab8295; RRID:AB_306445
Monoclonal mouse anti-Cdx2	BioGenex	Cat#AM392; RRID:AB_2650531
Monoclonal mouse anti-Esrrb	Perseus Proteomics	Cat#PP-H6705-00; RRID:AB_2100412
Polyclonal goat anti-FoxA2	R&D Systems	Cat#AF2400; RRID:AB_2294104
Polyclonal goat anti-Gata4	Santa Cruz Biotechnology	Cat#sc1237; RRID:AB_2108747
Polyclonal goat anti-Gata6	R&D Systems	Cat#AF1700; RRID:AB_2108901
Monoclonal rat anti-GFP	Nacalai Tesque	Cat#04404-84; RRID:AB_10013361
Monoclonal mouse anti-Klf2	Yamane et al., 2018	N/A
Rabbit serum anti-Klf2	Yeo et al., 2014	N/A
Polyclonal goat anti-Klf4	R&D Systems	Cat#AF3158; RRID:AB_2130245
Monoclonal rat anti-Nanog	eBioscience	Cat#14-5761-80; RRID:AB_763613
Polyclonal goat anti-Oct4	Santa Cruz Biotechnology	Cat#sc-8628; RRID:AB_653551
Monoclonal mouse anti-Oct4	Santa Cruz Biotechnology	Cat#sc-5279; RRID:AB_628051
Monoclonal rabbit anti-Oct4	Cell Signaling Technology	Cat#83932; RRID:AB_2721046
Monoclonal rabbit anti-Phospho-Smad1/5 (Ser463/465)	Cell Signaling Technology	Cat#13820; RRID:AB_2493181
Monoclonal rabbit anti-Phospho-Stat3 (Tyr705)	Cell Signaling Technology	Cat#9145; RRID:AB_2491009
Polyclonal goat anti-Sox1	R&D Systems	Cat#AF3369; RRID:AB_2239879
Monoclonal rat anti-Sox2	eBioscience	Cat#14-9811-80; RRID:AB_11219070
Polyclonal goat anti-Sox17	R&D Systems	Cat#AF1924; RRID:AB_355060
Polyclonal goat anti-T (Brachyury)	R&D Systems	Cat#AF2085; RRID:AB_2200235
Polyclonal goat anti-Tfcp2l1	R&D Systems	Cat#AF5726; RRID:AB_2202564
Monoclonal mouse anti-alpha-Tubulin	Abcam	Cat#ab7291; RRID:AB_2241126

**Chemicals, Peptides, and Recombinant Proteins**		

N2	Made in house	N/A
B27	GIBCO	Cat#17504-044
DMEM/F-12	GIBCO	Cat#21331-020
Neurobasal	GIBCO	Cat#21103-049
L-Glutamine	GIBCO	Cat#25030-024
2-mercaptoethanol	GIBCO	Cat#31350-010
Penicillin-streptomycin	Sigma-Aldrich	Cat#P0781
GSK3 inhibitor CHIR99021	ABCR	Cat#AB 253776
MEK inhibitor PD0325901	ABCR	Cat#AB 253775
LIF	Made in house	https://qkine.com/
Fgf2	Made in house	https://qkine.com/
ActivinA	Made in house	https://qkine.com/
XAV 939	Tocris	Cat#3748
Gelatin	Sigma-Aldrich	Cat#G1890
Fibronectin	Millipore	Cat#FC010
Accutase	Biolegend	Cat#423201
Lipofectamine-2000	Invitrogen	Cat#11668-030
Lipofectamine RNAiMAX	Invitrogen	Cat#13778-030
Hygromycin-B	ThermoFisher	Cat#10687010
Puromycin	ThermoFisher	Cat#A1113803
Blasticidin	Millipore	Cat#203351
G418	Invitrogen	Cat#10131019
Doxycycline	MP Biomedicals	Cat#198955
GCSF	Peprotech	Cat#300-23
BMP4	Miltenyi Biotec	Cat#130-098-787
DMH2	Tocris	Cat#5580
LDN193189	Sigma-Aldrich	Cat#SML0559
InSolution JAK Inhibitor I	Millipore	Cat#420097
M2 medium	Sigma-Aldrich	Cat#M7167
Blast medium	Origio	Cat#83060010
Cleav medium	Origio	Cat#83040010
Anti-mouse serum	Sigma-Aldrich	Cat#M5774
Non-heat-inactivated rat serum	Made in house	N/A
FCS	Labtech	Cat#FB-1001S/500
Trypsin	Life Technologies	Cat#25200072
5-Azacytidine	Sigma-Aldrich	Cat#A2385
IL6	Peprotech	Cat#200-06-20
Soluble IL6R	Peprotech	Cat#200-06R-20

**Critical Commercial Assays**		

RNeasy Kit	QIAGEN	Cat#74106
DNase I	QIAGEN	Cat#79254
SuperscriptIII VILO cDNA Synthesis Kit	Invitrogen	Cat#11754-250
TaqMan Fast Universal PCR Master Mix	Applied Biosystems	Cat#4352042
Fast SYBR Green Master Mix	Applied Biosystems	Cat#4385614

**Deposited Data**		

Single-cell RNA-seq data	This study	ArrayExpress: E-MTAB-7901
Bulk RNA-seq data	This study	ArrayExpress: E-MTAB-8046

**Experimental Models: Cell Lines**		

*Rex1*^*+/dGFP.IRES.bsd*^ EpiSCs	This study	N/A
*Rex1*^*+/dGFP.IRES.bsd*^ nPSCs	Kalkan et al., 2017	N/A
*Oct4*^*-/βgeo*^ CAG.Oct4^wt^.2A.mCherry nPSCs	Radzisheuskaya et al., 2013	N/A
*Oct4*^*-/βgeo*^ CAG.Oct4^wt^.2A.mCherry EpiSCs	This study	N/A
*Oct4*^*-/βgeo*^ CAG.Oct4^wt^.2A.mCherry MEFs	This study	N/A
*T*^*+/GFP*^*Rex1*^*+/mKO2.IRES.bsd*^ EpiSCs	This study, based on *T*^*+/GFP*^ nPSCs from Fehling et al. (2003)	N/A
*Gata6*^*+/H2BVenus*^ EpiSCs	This study, based on *Gata6*^*+/H2BVenus*^ nPSCs from Freyer et al. (2015)	N/A

**Experimental Models: Organisms/Strains**		

*Mus musculus* strain 129 was used to provide embryos for study and for ESC derivation	N/A	N/A
*Mus musculus* strain C57BL/6 was used to provide host embryos for chimeras	N/A	N/A

**Oligonucleotides**		

Esrrb FlexiTube GeneSolution siRNA	QIAGEN	Cat#1027416_ID: 26380
Klf2 FlexiTube GeneSolution siRNA	QIAGEN	Cat#1027416_ID:16598
Oct4 FlexiTube GeneSolution siRNA	QIAGEN	Cat#1027416_ID:18999
AllStars Negative Control siRNA	QIAGEN	Cat#1027281
RT-qPCR TaqMan probes	Applied Biosystems	See Table S2
Primers	Various	See Table S3

**Recombinant DNA**		

PB.TetO.Esrrb.PGK.hph	This study	N/A
PB.TetO.Klf2.PGK.hph	This study	N/A
PB.TetO.Klf4.PGK.hph	This study	N/A
PB.TetO.Klf5.PGK.hph	This study	N/A
PB.TetO.Nanog.PGK.hph	This study	N/A
PB.TetO.Tfcp2l1.PGK.hph	This study	N/A
PB.CAG.rtTA3.PGK.pac	This study	N/A
PB.CAG.GY118F.PGK.hph	This study	N/A
PB.CAG.GY118F.PGK.bsd	This study	N/A
Rex1-mKO2 fusion cassette	This study	N/A

**Software and Algorithms**		

Fiji	Schindelin et al., 2012	https://imagej.net/Fiji
R	The R Project	https://www.r-project.org
FlowJo	FlowJo, LLC	https://www.flowjo.com
Imaris	Oxford Instruments	https://imaris.oxinst.com/
CellProfiler	Carpenter et al., 2006	https://cellprofiler.org/
STAR	Dobin et al., 2013	https://github.com/alexdobin/STAR
Picard	Broad Institute	https://broadinstitute.github.io/picard
SAMtools	Li et al., 2009	http://www.htslib.org
HTSeq-count	Anders et al., 2015	https://htseq.readthedocs.io
DESeq2	Love et al., 2014	https://bioconductor.org/packages/release/bioc/html/DESeq2.html
DeconRNASeq	Gong & Szustakowski, 2013	https://www.bioconductor.org/packages/release/bioc/html/DeconRNASeq.html
scde	Kharchenko et al., 2014	https://hms-dbmi.github.io/scde
FactoMineR	Lê et al., 2008	http://factominer.free.fr
sincell	Juliá et al., 2015	http://bioconductor.org/packages/release/bioc/html/sincell.html
Rtsne	Krijthe, 2015	https://cran.r-project.org/web/packages/Rtsne
MFuzz	Kumar and E Futschik, 2007	https://www.bioconductor.org/packages/release/bioc/html/Mfuzz.html
gplots	Comprehensive R Archive Network (CRAN)	https://cran.r-project.org/web/packages/gplots

**Other**		

Single-cell RNA-seq data (E3.5, E4.5, E6.5 embryos)	Mohammed et al., 2017	GEO: GSE100597
Single-cell RNA-seq data (compacted morula)	Deng et al., 2014	GEO: GSE45719
Mouse reference genome NCBI build 38, GRCm38	Genome Reference Consortium	https://www.ncbi.nlm.nih.gov/grc/mouse
Ensembl release 87	EMBL-EBI	http://www.ensembl.org/

### Lead Contact and Materials Availability

Further information and requests for resources and reagents should be directed to and will be fulfilled by the Lead Contact, José Silva (jcs64@cam.ac.uk).

### Experimental Model and Subject Details

#### Mice

Mice used in this study were adult females aged 6-10 weeks. *Mus musculus* strain 129 was used to provide embryos for study and for ESC derivation. *Mus musculus* strain C57BL/6 was used to provide host embryos for chimeras. Work was performed in a UK Home Office designated facility in accordance with EU guidelines for the care and use of laboratory animals, and under authority of a UK Home Office project license. Use of animals in this project was approved by the Animal Welfare and Ethical Review Body for the University of Cambridge.

#### Cell lines

Murine naive pluripotent stem cells (nPSCs), murine epiblast stem cells (EpiSCs) and murine embryonic fibroblasts (MEFs) were employed for this study. nPSCs and EpiSCs were used from passage 10–25, and MEFs from passage 3–5. Culture conditions are detailed below. Cell lines were routinely tested and confirmed negative for mycoplasma.

### Method Details

#### Cell culture

nPSCs and inPSCs were cultured in N2B27+2i+LIF (2iLIF). EpiSCs were cultured in N2B27+ XAV+FGF2+ActivinA (FA). N2B27 medium comprised 1:1 DMEM/F-12 and Neurobasal (GIBCO), 2 mM L-glutamine (GIBCO), 1x penicillin-streptomycin (Sigma), 0.1 mM 2-mercaptoethanol (GIBCO), 1% B27 (GIBCO) and 0.5% N2 (homemade). As required, N2B27 was supplemented with 20 ng/ml murine LIF (homemade), 3 μM CHIR99021 (Chiron; CH) and 1 μM PD0325901 (PD03; PD) (ABCR), 12.5 ng/ml FGF2 and 20 ng/ml ActivinA (homemade), 6.25 μg/ml XAV 939 (Tocris), 3 μM DMH2 (Tocris), or 0.6 μM LDN193189 (Sigma). For nPSCs and inPSCs, tissue-culture flasks were coated with 0.15% gelatin (Sigma) in PBS (Sigma) and incubated in 7% CO_2_. For EpiSC culture and reprogramming experiments, tissue-culture flasks were coated with 10 μg/ml fibronectin (Millipore) in PBS (Sigma) and incubated in 7% CO_2_ and 5% O_2_. nPSCs, inPSCs and EpiSCs were dissociated with accutase (Biolegend) during passaging. For optimal performance of EpiSCs, lines were maintained by plating 25000 cells/cm^2^ every other day (usually 1:6 split ratio) following gentle accutase treatment for less than 3 minutes at room temperature. For Figures S5F and S5G and 6D, nPSCs were cultured in FCS+LIF medium containing GMEM (Sigma), 10% fetal calf serum (FCS) (Labtech), 1x non-essential amino acids (GIBCO), 1 mM sodium pyruvate (Sigma), 2 mM L-glutamine (GIBCO), 1x penicillin-streptomycin (Sigma), 0.1 mM 2-mercaptoethanol (GIBCO), 20 ng/ml murine LIF (homemade), and 10 ng/ml BMP4 (Miltenyi Biotec) was supplemented as indicated.

#### Derivation of Rex1::dGFP EpiSCs

*Rex1*^*dGFP.IRES.bsd/dGFP.IRES.bsd*^ homozygous 129 studs (Kalkan et al., 2017) were crossed with wild-type 129 females and heterozygous *Rex1*^*+/dGFP.IRES.bsd*^ EpiSCs (referred to as *Rex1::*dGFP reporter) were derived from resultant E6.5 embryos. Epiblasts were manually dissected from extra-embryonic tissues and plated on fibronectin-coated plates in FA medium. After 5–7 days of culture, regions of the explant exhibiting EpiSC morphology were manually passaged to a fresh plate. Subsequent passages were performed using accutase.

#### Cell transfection

For transgene integration transfections, 1 μg PiggyBac (PB) vectors of interest, 0.5 μg PBase expression vector (*CAG.PBase*) and 10 μL Lipofectamine-2000 (Invitrogen) were incubated for 20 min in 500 mL DMEM (GIBCO), then applied to 500,000 cells/6well in 3 mL medium for 18 hours. Selection was applied to transfectants for at least 5 passages prior to use: 50 μg/ml hygromycin-B (ThermoFisher) for *PB.TetO.GOI.PGK.hph* or *PB.CAG.GY118F.PGK.hph*, and 0.33 μg/ml puromycin (ThermoFisher) for *PB.CAG.rtTA3.PGK.pac*. siRNA transfections were performed using RNAiMAX transfection reagent (Invitrogen) and FlexiTube siRNAs against Oct4, Klf2, Esrrb, or AllStars Negative Control (QIAGEN) according to the manufacturers’ instructions.

#### EpiSC reprogramming

EpiSCs were plated in FA without selection at a density of 2000/24well or equivalent. For siRNA experiments, 10000/24well or equivalent was used instead to compensate for transfection toxicity. The following day, reprogramming was induced by medium change to 2iLIF or subset components thereof as indicated, together with driver induction as appropriate. Expression of TetO transgenes was induced with 1 μg/ml doxycycline (dox) (MP Biomedicals). GY118F transgenic receptor (iPStat3) was stimulated with 30 ng/ml human GCSF (Peprotech). After 4 days, transgene induction was withdrawn and 20 μg/ml blasticidin (Millipore) was applied to select for *Rex1::*dGFP.IRES.bsd activity. On day 8, 4x images were acquired using CellSens software and an X-51 Olympus microscope system with motorized stage and camera. inPSC colonies with active *Rex1* reporter were counted manually. *Rex1* reporter activity confers both dGFP expression and blasticidin resistance, and is a well-characterized naive marker (Kalkan et al., 2017). We confirmed that dGFP+ colonies are also Oct4+Tfcp2l1+ by immunostaining (data not shown). Only 4 days of transgene induction is a stringent test of driver efficacy; we note that more colonies emerged when induced for longer, including for weaker drivers iNanog and iTfcp2l1 (data not shown). No inPSC colonies ever emerged from any EVrtTA3+dox nor EV+GCSF reprogramming experiments, confirming that our lines represent ‘late-stage’ EpiSCs (Han et al., 2010). Where indicated, reprogramming experiments were treated with 3 μM DMH2 (Tocris), 0.6 μM LDN (Sigma), or 1 μM Jak inhibitor (Millipore) from days 0–4. Unless stated otherwise, reprogramming data presented are the mean of 3 biological replicates.

#### T::GFP Rex1::mKO2 EpiSCs

*T*^*+/GFP*^ nPSCs (Fehling et al., 2003) were kindly shared by Gordon Keller. Rex1-mKO2 fusion cassette was constructed by replacing the dGFP cassette of the Rex1-dGFP targeting vector (Kalkan et al., 2017, Wray et al., 2011) (Figure S3B). We linearized the vector with BspH1, then electroporated it into *T*^*+/GFP*^ nPSCs using Gene Pulser (BioRad) at 230V, 500μF. Correct targeting results in a Rex1-mKO2 fusion protein and confers blasticidin resistance when *Rex1* is expressed. nPSCs were selected with 10 μg/ml blasticidin, then clones were genotyped by PCR. PCR primers for 5′ side are TCGTGTGACTCTGCATCTGT and CTGCCTCTTTAGCTGCGG, and for 3′ side are ATTCGTGAATTGCTGCCCTC and GAGGCAGAGGAACAGGACTT. Correctly targeted nPSC clone TGHRO6 (subsequently referred to as TGRO) was differentiated to EpiSCs by 10 passages in FA, resulting in *T::*GFP *Rex1::*mKO2 double-reporter EpiSCs.

#### Gata6::H2BVenus EpiSCs

*Gata6*^*+/H2BVenus*^ nPSCs (Freyer et al., 2015) were kindly shared by Christian Schröter. By differentiation for 10 passages in FA, we obtained *Gata6::*H2BVenus reporter EpiSCs.

#### Live imaging

Live imaging was performed using IncuCyte system, with phase and H2BVenus images taken every 60 min for *Gata6* reporter, or phase, GFP and mKO2 images taken every 45 min for *T/Rex1* double-reporter. For *Gata6* reporter, the endpoint was fixed, stained for Tfcp2l1 (AF594) and Oct4 (AF647), then re-imaged with the same positional registration for AF594. Co-expression of Tfcp2l1+Oct4+ in endpoint inPSCs was confirmed on a separate microscope capable of detecting AF647 as well (data not shown).

#### FixedOct4 EpiSCs

FixedOct4 EpiSCs were generated from *Oct4*^*-/βgeo*^ CAG.Oct4^wt^.2A.mCherry nPSCs (Radzisheuskaya et al., 2013) by differentiation in FA for 10 passages. *Oct4*^*F/βgeo*^ CAG.EmptyVector EpiSCs were generated as a control from the same parental line. Reprogramming was conducted in N2B27+2iLIF as above. 200 μg/ml G418 (Invitrogen) was applied to select for endogenous *Oct4* promoter activity after 4 days of reprogramming.

#### Reprogramming from FixedOct4 E9.5 chimeras

E9.5 chimeras were generated by blastocyst injection of *Oct4*^*-/βgeo*^ CAG.Oct4^wt^.2A.mCherry nPSCs (Radzisheuskaya et al., 2013). The tail portions of resulting E9.5 embryos were removed to strictly avoid germ cell contamination in the cultures. The anterior portion was dissociated manually, then subdivided into quarters (2x LIFaza, 2x 2iLIF or LIF only as indicated). Aza = 1 μM 5-Azacytidine (Sigma). After 6 days, LIF or LIFaza was exchanged for 2iLIF, then on day 10 G418 was applied to all cultures (200 μg/ml). Chimeric allantois samples were dissociated manually, a portion taken for expression analysis, and the remainder plated as positive control (germ-cell containing).

#### Derivation and reprogramming of FixedOct4 MEFs

E12.5 chimeras were generated by blastocyst injection of *Oct4*^*-/βgeo*^ CAG.Oct4^wt^.2A.mCherry nPSCs (Radzisheuskaya et al., 2013). The heads and all internal organs were removed, taking particular care to fully remove the genital ridges. Carcasses were dissociated in trypsin (Life Technologies), then cultured in FCS medium. Hygromycin was applied to select for CAG.Oct4^wt^.2A.mCherry transgene. MEFs were passaged using trypsin, and used from passage 3–5 for reprogramming assays. For reprogramming, MEFs were plated at 5000/24well in FCS medium on 0.15% gelatin. The following day, medium was changed to N2B27+LIF ± IL6&IL6R ± FGF2 ± CH ± PD as indicated for the first week (20 ng/ml LIF; 50 ng/ml IL6 and 10 ng/ml soluble IL6R; 12.5 ng/ml FGF2; 3 μM CH; 1 μM PD). On day 7, all were swapped to N2B27+2iLIF, then on day 14 G418 was added (200 μg/ml) to select for inPSC colonies. We would like to highlight that all medium was N2B27-based, i.e., MEF reprogramming occurred in the absence of serum, KSR/ascorbic acid, or any small molecule epigenetic modulators.

#### Microinjection to generate chimeras

Chimeras were generated from strain 129 (agouti) male inPSCs by standard microinjection methodology using host blastocysts of strain C57BL/6 (black), followed by gestation in pseudo-pregnant recipient females. Germline-competence of male chimeras was tested by crossing them to C57BL/6 (black) females and checking for agouti pups. For Figures 4G–4I, host embryos were injected at the 8-cell stage, cultured in Blast medium (Origio) until blastocysts formed, then cultured to the late blastocyst stage in N2B27. For Figure S7E, tetraploid host embryos were generated by cell fusion at the 2-cell stage, cultured to the 8-cell stage in Cleav medium (Origio), injected then transferred to pseudo-pregnant recipients for gestation.

#### BMP inhibitor treatment of embryos

Wild-type 129 mice were crossed and embryos flushed from oviducts at 2.75 dpc using M2 medium (Sigma). Embryos were subsequently incubated in Blast medium (Origio) and periodically inspected. At cavitation onset, embryos were randomly divided into Blast medium supplemented either with 3 μM DMH2 (Tocris), 0.3 μM LDN (Sigma) or 1:1000 DMSO. Once blastocysts had fully formed, they were transferred to N2B27 medium continuing DMH2/LDN/DMSO treatment as before. At the late blastocyst stage, embryos were fixed and immunostaining was performed. Embryos were permeabilized in 0.25% Triton X-100 (Sigma) in PBS for 30 min, then blocked in 3% donkey serum (Sigma), 0.1% BSA (Sigma) and 0.01% Tween-20 (Sigma) for 30 min at room temperature. Embryos were incubated overnight at 4°C in blocking buffer with the following primary antibodies: Cdx2 (1:500, mouse mAb, BioGenex); Gata4 (1:300, goat pAb, Santa Cruz); Nanog (1:300, rat mAb, eBioscience); Oct4 (1:300, rabbit mAb, Cell Signaling). The following day, washes were performed in blocking buffer. AlexaFluor secondary antibodies (Life Technologies or Abcam) were used against the appropriate species at 1:1000 in blocking buffer. Embryos were gradually acclimatised then mounted in Fluoromount-G (Southern Biotech) and images were taken with a Zeiss 710 LSM confocal microscope. Presented images are maximum intensity projections of Z stack slices processed with ImageJ. Staining quantification was carried out with Imaris: nuclei were identified in the DAPI channel and the fluorescence of each other channel recorded. Cells were assigned to each lineage based on position and marker staining. We note that the phenotype was highly time-sensitive: addition of BMP inhibitor at the 8-cell stage caused developmental arrest, consistent with Reyes de Mochel et al., 2015 but precluding fair assessment of whether the naive lineage specifically is compromised. At the 8-cell stage, the trophectoderm (TE) versus inner cell mass (ICM) decision has not yet been made. Conversely, application of BMP inhibitor to the mid blastocyst did not disrupt the naive epiblast (data not shown). By this point, the epiblast versus primitive endoderm (PrE) bifurcation is already underway. In contrast, precisely timed inhibitor addition at cavitation onset in the late morula falls between these two developmental lineage bifurcations, and thus permits assessment of the role of BMP signaling in naive epiblast establishment despite the multitude of BMP signaling roles during early development.

#### Quantitative nPSC derivation

Following BMP inhibitor treatment from cavitation onset as above, quantitative nPSC-derivation was performed from late blastocysts as previously described (Nichols et al., 2009). Briefly, immunosurgery was performed to remove the TE using anti-mouse serum (Sigma) then non-heat-inactivated rat serum as complement (homemade). Then, the ICM (comprising epiblast+PrE) was dissociated to single cells using accutase. 10 single cells were manually transferred to each 96well and cultured in feeder-free N2B27+2iLIF conditions on gelatin, without any further DMSO/DMH2 treatment so that we could assess whether the epiblast of the embryo was already affected. The number of nPSC colonies was scored after 6 days, and nPSC identity confirmed by RT-qPCR (data not shown).

#### Flow cytometry

Flow cytometry was performed using a BD LSRFortessa analyzer with subsequent data analysis using FlowJo software. Cell sorting was performed using a MoFlo Legacy Cell Sorter (Beckman) or an S3 Cell Sorter (BioRad). dGFP was excited using a 488 nm laser and detected using a 530/30 filter. *Rex1::dGFP* EpiSCs and nPSCs were used to determine negative and positive dGFP gates respectively. After sorting of reprogramming intermediates, number of inPSC colonies are quantified relative to the number of nPSC colonies, because replating of sorted nPSCs provides a control for cell death due to the stress of sorting. nPSCs already stably occupy the destination naive pluripotent identity, and are thus the appropriate functional control. When we replated *Rex1::*dGFP+ reprogramming intermediates for clonogenicity assay, we later applied blasticidin as an additional control to prove that the *Rex1* promoter was active in scored inPSC colonies: *Rex1* promoter drives both dGFP expression and blasticidin resistance.

#### Immunohistochemistry

Cultured cells were fixed with 4% paraformaldehyde (Sigma) in PBS for 10 min at room temperature. E9.5 embryos were fixed with 4% paraformaldehyde (Sigma) in PBS for 4 hours at 4°C, gradually adjusted to 20% sucrose over 2 days, mounted in O.C.T. (TissueTek), snap frozen on liquid nitrogen, cryosectioned (8 μm), stored at −80°C, then rehydrated in PBS. For both cultured cells and embryo cryosections, permeabilization was performed in 0.4% Triton X-100 (Sigma) in PBS, then samples were blocked in 5% donkey serum (Sigma) and 0.1% Triton X-100 in PBS. Samples were incubated overnight at 4°C in blocking buffer with the following primary antibodies: Cardiac troponin (1:300, mouse mAb, Abcam); Esrrb (1:300, mouse mAb, Perseus Proteomics); FoxA2 (1:300, goat pAb, R&D); Gata4 (1:300, goat pAb, Santa Cruz); Gata6 (1:300, goat pAb, R&D); GFP (1:300, rat mAb, Nacalcai); Klf2 (kind gift from Hitoshi Niwa; 1:300, mouse mAb, Yamane et al., 2018); Klf4 (1:300, goat pAb, R&D); Nanog (1:300, rat mAb, eBioscience); Oct4 (1:300, rabbit mAb, Cell Signaling); Oct4 (1:100, mouse mAb, Santa Cruz); PSmad1/5 (1:100, rabbit mAb, Cell Signaling); P-Y705-Stat3 (1:300, rabbit mAb, Cell Signaling); Sox1 (1:300, goat pAb, R&D); Sox2 (1:300, rat mAb, eBioscience); Sox17 (1:300, goat pAb, R&D); T (1:300, goat pAb, R&D); Tfcp2l1 (1:300, goat pAb, R&D). The next day, washes were performed with 0.1% Triton X-100 in PBS, and samples incubated with DAPI (ThermoFisher) and AlexaFluor secondary antibodies against the appropriate species at 1:1000 (Life Technologies). For PSmad1/5 and PStat3 stainings, TBS was used at all steps instead of PBS. Samples were mounted in Fluoromount-G (Southern Biotech) and imaged using Leica DMI6000, Nikon Eclipse Ti Spinning Disk confocal or Zeiss ApoTome microscope. Staining quantification was carried out with CellProfiler: nuclei were identified in the DAPI channel and the fluorescence of each other channel recorded. Confocal images are presented as maximum intensity projections of Z stack slices processed with ImageJ. Embryo sections were imaged using the Zeiss ApoTome microscope at 20x then tiled. After imaging, H&E histological staining was performed on the same sections according to standard methodologies. These sections were then re-imaged in the same pipeline.

#### Western blotting

Cells were lysed in RIPA buffer (Sigma) containing Complete-ULTRA protease-inhibitor and PhoStop phosphatase-inhibitor cocktails (Roche), and sonicated with Bioruptor200 (Diagenode) at high frequency, alternating 30 s on/off for 3 min. SDS-PAGE electrophoresis was performed using Bolt 10% Bis-Tris Plus gels (ThermoFisher) in a Novex MiniCell (ThermoFisher). Protein transfer was performed using the semi-dry iBlot2 system (ThermoFisher) and iBlot Transfer Stacks (ThermoFisher). The following primary antibodies were used: Esrrb (1:1000, mouse mAb, Perseus Proteomics); Klf2 (kind gifts from Huck-Hui Ng; 1:500, rabbit serum, Yeo et al., 2014; and Hitoshi Niwa (1:1000, mouse mAb; Yamane et al., 2018); Oct4 (1:1000, rabbit mAb, Cell Signaling); P-Y705-Stat3 (1:1000, rabbit mAb, Cell Signaling); αTubulin (1:10000, mouse mAb, Abcam). Detection was achieved using HRP-linked secondary antibodies at 1:10000 against the appropriate species (GE Healthcare) and ECL Plus Western Blotting Detection System (GE Healthcare).

#### Genotyping

Genotyping to distinguish between *Oct4*^*-/βgeo*^, *Oct4*^*F/βgeo*^ and *Oct4*^*+/+*^ cells was conducted using Taq DNA Polymerase (QIAGEN) according to manufacturer’s instructions and the following thermocycler program: 95°C 3 min; 30x 94°C 15 s, 60°C 30 s, 72°C 60 s; 72°C 10 min. Primer GAGCTTATGATCTGATGTCCATCTCTGTGC binds in the *Oct4* final intron, which is present in both wild-type and Flox (F) alleles. Primer GGGCTGACCGCTTCCTCGTGCTTTACG binds in the *βgeo* allele. Primer GCCTTCCTCTATAGGTTGGGCTCCAACC binds 3′ downstream of Oct4 and is common to all alleles.

#### Plasmids

PiggyBac expression vector was modified to contain TetO rather than CAG promoter. Genes of interest (Esrrb, Klf2, Klf4, Klf5, Nanog, Tfcp2l1) were cloned into the resultant vector using Gateway technology (ThermoFisher) according to manufacturer’s instructions, producing inducible expression vectors in the form *PB.TetO.GOI.PGK.hph*. GY118F and rtTA3 expression vectors were generated in the same manner, but retaining CAG instead of TetO promoters and thus yielding *PB.CAG.GY118F.PGK.hph* and *PB.CAG.rtTA3.PGK.pac* respectively. *PB.CAG.GY118F.PGK.bsd* was used in the *Gata6::*H2BVenus reporter line.

#### RT-qPCR

Total RNA was extracted using RNeasy kits, according to manufacturer’s spin protocol, including on-column DNaseI digest (QIAGEN). cDNA was produced from 1 μg RNA using SuperscriptIII VILO cDNA synthesis kit, following the recommended protocol (Invitrogen). RT-qPCR reactions were performed using StepOnePlus Real Time PCR System with recommended thermocycler settings (Applied Biosystems) and TaqMan Fast Universal PCR Master Mix (Applied Biosystems). Gene expression relative to Gapdh in each well was determined using VIC-labeled Gapdh probe 4352339E together with FAM-labeled TaqMan assay probe (Applied Biosystems): Esrrb Mm00442411_m1; Fgf5 Mm00438918_m1; Gata6 Mm00802636_m1; Klf2 Mm01244979_g1; Klf4 Mm00516104_m1; Klf5 Mm00456521_m1; Nanog Mm02384862_g1; Oct4 (Pou5f1) Mm00658129_gH; Sox2 Mm03053810_s1; T (Brachyury) Mm01318252_m1; Tfcp2l1 Mm00470119_m1; Rex1 (Zfp42) Mm03053975_g1. RT-qPCR against Col1a1 and Prrx1 was performed using Fast SYBR Green Master Mix (Applied Biosystems) and the ΔΔCt method to calculate relative expression using Gapdh as a reference gene and MEFs derived from an mCherry-negative littermate as a reference sample. KiCqStart primers (Sigma) were used against Col1a1 (F GATCTGTATCTGCCACAATG, R TGGTGATACGTATTCTTCCG) or against Prrx1 (F GAAAAAGAACTTCTCCGTCAG, R CTTTCTCTTCTTCTTCTCCTC) at a final concentration of 450nM. Custom primers (Sigma) against Gapdh (F CCCACTAACATCAAATGGGG, R CCTTCCACAATGCCAAAGTT) were used at a final concentration of 450nM. In all cases, melt curves were performed to validate that there was no significant off-target amplification.

#### Bulk RNA-seq library preparation

Sequencing libraries were prepared according to the SmartSeq2 protocol (Picelli et al., 2014) with the following amendments: purified RNA was used diluted to 5 ng/μl; ERCC spike-ins (Invitrogen) were added at 1 μl of 1:10000 dilution per 5 ng; 13 cycles of amplification were used to obtain cDNA (rather than 21 cycles used for single-cells). Nextera XT reactions were scaled-down by half, using 0.4ng cDNA input per reaction. Pooled libraries were sequenced on the Illumina HiSeq 4000 (paired-end 150bp reads).

#### scRNA-seq library preparation

Single cells were index-sorted individually by FACS (BD Influx 5) into wells of a 96-well PCR plate containing lysis buffer. scRNA-seq was performed as previously described (Nestorowa et al., 2016, Picelli et al., 2014, Wilson et al., 2015) for a total of 1152 single cells. The Illumina Nextera XT DNA kit was used to prepare libraries. Pooled libraries were sequenced on the Illumina HiSeq 4000 (single-end 125bp reads). Samples from all cell lines were included in each sequencing lane, to control for technical lane effects. We did not detect a significant batch effect.

#### RNA-seq alignment and processing

Sequencing reads were aligned to mouse genome reference GRCm38/mm10 with STAR (Dobin et al., 2013) using the two-pass method for novel splice detection (Engström et al., 2013). GENCODE M12 mouse gene annotation from Ensembl release 87 (Yates et al., 2016) was used for read alignment and splice junction donor/acceptor overlap settings were tailored to the read length of each dataset. Alignments to gene loci were quantified with HTSeq-count (Anders et al., 2015) based on annotation from Ensembl release 87. Sequencing libraries with fewer than 500,000 mapped reads were excluded from subsequent analyses. Read distribution bias across gene bodies was computed as the ratio between the total reads spanning the 50^th^ to the 100^th^ percentile of gene length, and those between the first and 49^th^. Samples with ratio > 2 were not considered further.

#### Published embryo scRNA-seq datasets

Sequencing data from single-cell mouse embryo profiling studies SRP110669 (Mohammed et al., 2017; E3.5, E4.5, E6.5) and SRP020490 (Deng et al., 2014; compacted morula) were obtained from the European Nucleotide Archive (Toribio et al., 2017) and aligned as above.

#### Transcriptome analysis

Principal component and cluster analyses were performed based on log_2_ FPKM values and were computed with the Bioconductor packages DESeq2 (Love et al., 2014), Sincell (Juliá et al., 2015) or FactoMineR (Lê et al., 2008) in addition to custom scripts. Default parameters were used unless otherwise indicated. For global analyses, genes that registered zero counts in all single-cell samples were omitted. Euclidean distance and average agglomeration methods were used for cluster analyses unless otherwise indicated. t-SNE analysis was computed using Rtsne R package (Krijthe, 2015) with default parameters. *k*-means hard clustering was performed using the Mfuzz R package (Kumar and E Futschik, 2007) and the optimal number of *k* clusters were selected using the elbow method. The gplots R package was used to generate heatmaps.

#### Selection of high-variability genes

Genes exhibiting the greatest expression variability (and thus contributing substantial discriminatory power) were identified by fitting a non-linear regression curve between average log_2_ FPKM and the square of coefficient of variation. Indicated specific thresholds were applied along the x axis (average log_2_ FPKM) and y axis (CV^2^) to identify the most variable genes.

#### Quadratic programming

Fractional identity of single cells was computed using R package DeconRNASeq (Gong & Szustakowski, 2013). This package utilizes quadratic programming to estimate the proportion of distinctive cell types. The average expression of bulk RNA-seq samples (EpiSCs or inPSCs, Figure S2C) or the average expression of scRNA-seq samples (embryo stages; Figure 4B) were used as signature datasets. The fraction of identity between single cells and the signature datasets was computed using the whole transcriptome. Reprogramming pseudotimes for single cells were assigned by ordering cells based on their fraction of similarity to EpiSCs (origin) and inPSCs (destination) (Figure S2C), as previously described (Treutlein et al., 2016).

#### LOESS regression

Smooth curve local regression (LOESS) lines were fitted to scatterplots of log_2_ FPKM versus pseudotime using the R stats package (R Core Team, 2016). Smoothness parameter of 1/3 and 2 degrees of local polynomial were used for curve fitting.

#### Differential gene expression

Differential expression (DE) analysis was performed on each sample set relative to start EpiSCs, using the R package scde (Kharchenko et al., 2014) which fits individual error models for assessment of differential expression between groups of single cells.

### Quantification and Statistical Analysis

Where appropriate, two-sided t tests were performed and *p-value*s indicated on the Figures. Error bars on Figures represent ± one standard deviation (SD), and n is noted on each Figure or in the corresponding Figure Legend.

### Data and Code Availability

The single-cell and bulk RNA-seq data generated during this study are available in the ArrayExpress repository under accessions E-MTAB-7901 and E-MTAB-8046, respectively.

## Supporting Citations

The following references appear in the Supplemental Information: Arai et al. (2001); Artus et al. (2011); Boroviak et al. (2015); Le Bin et al. (2014); and Silva et al. (2009).

## Supplemental Information

Document S1. Figures S1–S7 and Tables S1–S3

Document S2. Article plus Supplemental Information
