# Supplementary material for: Distinct Molecular Trajectories Converge to Induce Naive Pluripotency
Source: Cell Stem Cell. 2019 Sep 5;25(3):388–406.e8. doi: 10.1016/j.stem.2019.07.009 (PMC6731995; doi:10.1016/j.stem.2019.07.009)
Supplement: Document S1. Figures S1–S7 and Tables S1–S3 [file mmc1.pdf]

Cell Stem Cell, Volume *25*

Supplemental Information

Distinct Molecular Trajectories Converge to Induce Naive Pluripotency

Hannah T. Stuart, Giuliano G. Stirparo, Tim Lohoff, Lawrence E. Bates, Masaki Kinoshita, Chee Y. Lim, Elsa J. Sousa, Katsiaryna Maskalenka, Aliaksandra Radzisheuskaya, Andrew A. Malcolm, Mariana R.P. Alves, Rebecca L. Lloyd, Sonia Nestorowa, Peter Humphreys, William Mansfield, Wolf Reik, Paul Bertone, Jennifer Nichols, Berthold Göttgens, and José C.R. Silva

Figure S1

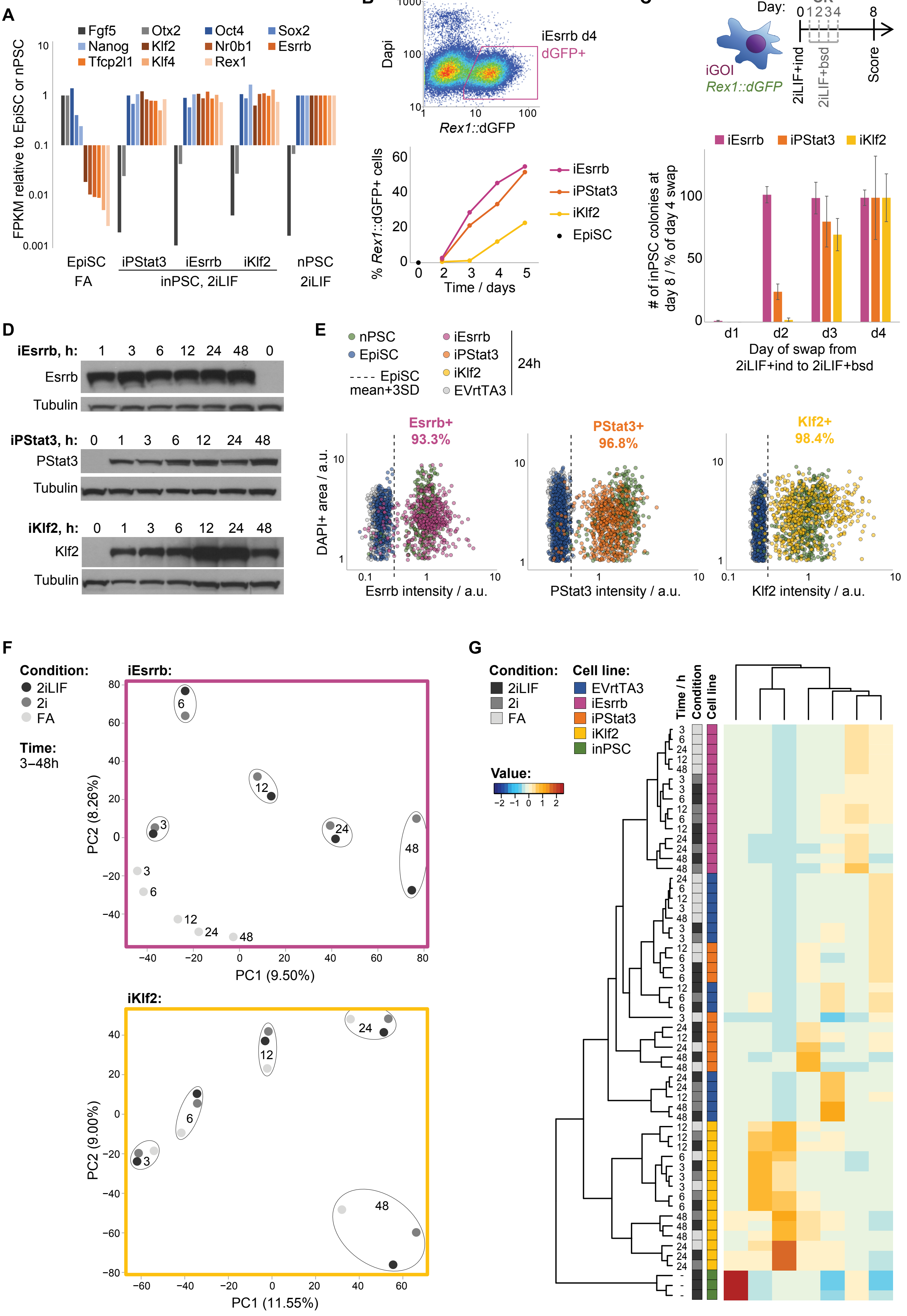

A
Fgf5 Otx2 Oct4 Sox2
Nanog Klf2 Nr0b1 Esrrb
Tfcp2l1 Klf4 Rex1
FPKM relative to EpiSC or nPSC
EpiSC FA
iPStat3 iEsrrb iKlf2
inPSC, 2iLIF
nPSC 2iLIF
B
Dapi
iEsrrb d4
dGFP+
Rex1::dGFP
% Rex1::dGFP+ cells
Time / days
iEsrrb
iPStat3
iKlf2
EpiSC
C
Day:
OR
iGOI
Rex1::dGFP
2iLIF+ind
2iLIF+bsd
Score
iEsrrb iPStat3 iKlf2
# of inPSC colonies at day 8 / % of day 4 swap
d1 d2 d3 d4
Day of swap from 2iLIF+ind to 2iLIF+bsd
D
iEsrrb, h: 1 3 6 12 24 48 0
Esrrb
Tubulin
iPStat3, h: 0 1 3 6 12 24 48
PStat3
Tubulin
iKlf2, h: 0 1 3 6 12 24 48
Klf2
Tubulin
E
nPSC
EpiSC
---- EpiSC mean+3SD
iEsrrb
iPStat3
iKlf2
EVrtTA3
24h
Esrrb+ 93.3%
PStat3+ 96.8%
Klf2+ 98.4%
DAPI+ area / a.u.
Esrrb intensity / a.u.
PStat3 intensity / a.u.
Klf2 intensity / a.u.
F
Condition:
2iLIF
2i
FA
Time:
3–48h
iEsrrb:
PC2 (8.26%)
PC1 (9.50%)
iKlf2:
PC2 (9.00%)
PC1 (11.55%)
G
Condition:
2iLIF
2i
FA
Cell line:
EVrtTA3
iEsrrb
iPStat3
iKlf2
inPSC
Value:
Time / h
Condition
Cell line

Figure S1, related to Figure 1:
Reprogramming initiation is driver-dependent

(A) Gene expression of established inPSCs at passage 5, together with control EpiSCs and nPSCs. Means are shown from scRNA-seq. Greys: primed markers. Blues: core pluripotency markers. Oranges: naïve markers. Together with Fig 1E, this shows that inPSCs derived with each driver do not differ in their molecular signatures.

(B) Percentage of *Rex1::*dGFP+ cells is shown for each driver after reprogramming induction in 2iLIF+dox (iEsrrb, iKlf2) or 2iLIF+GCSF (iPStat3), from days 2–5. Note that in standard reprogramming assays we would normally withdraw dox/GCSF at day 4 and add blasticidin to select for *Rex1* reporter activity. However, in this instance we continued in 2iLIF+dox/GCSF until day 5 and never added blasticidin, so that the % of dGFP+ cells was not confounded. Parental *Rex1::*dGFP EpiSCs in FA (0.00% dGFP+) and *Rex1::*dGFP nPSCs in 2iLIF (99.50% dGFP+) provided negative and positive controls respectively. Example: FACS-plot showing emergence of *Rex1::*dGFP expression during reprogramming of iEsrrb in 2iLIF+dox at day 4.

(C) Reprogramming of iEsrrb, iPStat3 and iKlf2 EpiSCs was induced (ind) at day 0 in 2iLIF+dox (iEsrrb, iKlf2) or 2iLIF+GCSF (iPStat3). Transgene induction by dox/GCSF was withdrawn on either day 1, 2, 3 or 4, with concomitant addition of blasticidin (bsd) to select for *Rex1* reporter activity. Naïve colonies were scored on day 8, and are presented as mean ±SD (n=3), relative to day 4. iEsrrb is the fastest to yield transgene-independent inPSCs, while iKlf2 is the slowest, consistent with their differing rates of *Rex1::*dGFP induction (Fig S1B) and their differing kinetics of naïve gene expression induction (Fig 1G).

(D) Timecourse of driver protein induction from 0–48h. Western blots are shown against PStat3, Klf2 and Esrrb, with αTubulin providing loading control. Induction is robust from 1h onwards for all drivers. Therefore, differences in naïve network induction kinetics are due to the downstream responses, rather than due to delays in transgene induction itself.

(E) Immunofluorescent staining was performed and quantified 24h after transgene induction, on a total of 5956 cells. EpiSC and nPSC samples provide negative and positive controls respectively. To determine the % of driver-positive cells, a stringent threshold was calculated: the mean of EpiSC values plus three standard deviations (3SD), indicated on the plots. Quantification of driver proteins in single cells shows efficient inductions (93–98%) to expression levels comparable to those of the endogenous proteins in nPSCs, which is the biologically relevant reference. DAPI+ area is plotted on the *y*-axis simply to assist in data visualisation.

(F) RNA-seq was performed on bulk samples after driver induction in 2i±LIF and in FA. Principle component analyses (PCA) based on expressed genes (FPKM>0) are shown for iEsrrb (above) and iKlf2 (below).

(G) *k*-means clustering of bulk RNA-seq samples, based on expressed genes (FPKM>0). Optimal *k* cluster number was computed using the elbow method.

Figure S2

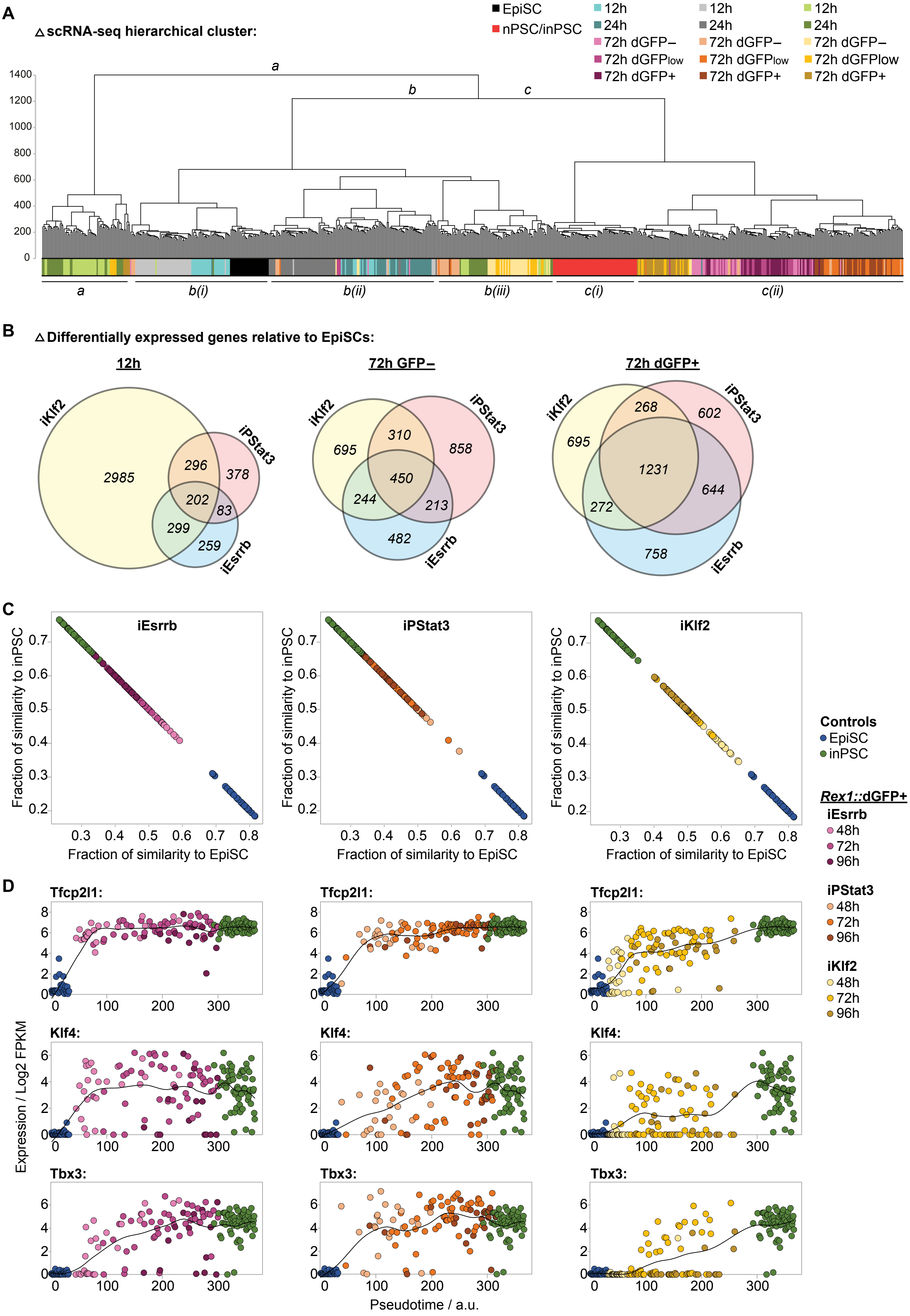

A
△scRNA-seq hierarchical cluster:
Controls
EpiSC
nPSC/inPSC
iEsrrb
12h
24h
72h dGFP–
72h dGFPlow
72h dGFP+
iPStat3
12h
24h
72h dGFP–
72h dGFPlow
72h dGFP+
iKlf2
12h
24h
72h dGFP–
72h dGFPlow
72h dGFP+
a
b
c
a
b(i)
b(ii)
b(iii)
c(i)
c(ii)
B
△Differentially expressed genes relative to EpiSCs:
12h
72h GFP–
72h dGFP+
iKlf2
iPStat3
iEsrrb
2985
296
378
202
83
299
259
695
310
858
450
244
213
482
268
602
1231
272
644
758
C
iEsrrb
iPStat3
iKlf2
Fraction of similarity to inPSC
Fraction of similarity to EpiSC
Controls
EpiSC
inPSC
Rex1::dGFP+
iEsrrb
48h
72h
96h
iPStat3
48h
72h
96h
iKlf2
48h
72h
96h
D
Tfcp2l1:
Klf4:
Tbx3:
Expression / Log2 FPKM
Pseudotime / a.u.

Figure S2, related to Figure 2:
Single-cell RNA-seq defines distinct productive trajectories

(A) Unsupervised hierarchical clustering, computed based on all genes with the Ward.D2 agglomeration method and Euclidean distances. iKlf2 12/24h cells cluster in a separate branch '*a*' from all other samples, indicating that they are more different to the rest than even EpiSC (start) vs nPSC/inPSC (end) samples are to each other. This is consistent with an initial diversion in the iKlf2 trajectory. The remainder of the samples clustered into indicated branches *b* and *c*. Branch *b* subclustered into: *b(i)*, comprised of EpiSCs and mostly 12h iPStat3 and iEsrrb; *b(ii),* mostly 24h iPStat3 and iEsrrb; *b(iii),* a mixture of early and mostly 72h dGFP– samples. Branch *c* subclustered into: *c(i)*, comprised of nPSC/inPSCs; and *c(ii)*, mostly 72h dGFPlow and dGFP+ samples for all drivers. Overall, this indicates that early timepoints and less productive populations are more similar to EpiSCs (start identity) whereas later and higher dGFP samples are more similar to nPSC/inPSCs (end identity). Importantly, within groups *b* and *c*, cells cluster according to driver rather than according to timepoint or dGFP status, demonstrating that routes are transcriptionally distinct throughout.

(B) Differential expression (DE) analysis was performed on each sample set relative to start EpiSCs. The resulting lists were compared and Venn diagrams plotted to find the numbers of DE genes that are unique to or shared between drivers at each timepoint. Examples of the Venn diagrams are shown here, and the DE numbers are summarised in main Fig 2E.

(C) Computation of fraction of similarity between each single cell vs EpiSCs in FA and inPSCs in 2iLIF. Signature EpiSC and inPSC datasets were generated by averaging of bulk RNA-seq samples. Single cells were ordered from lowest to highest inPSC/EpiSC identity fractions to generate pseudotime coordinates from reprogramming start to end. Pseudotime coordinates largely agreed with real-time.

(D) Scatter plots of expression in single cells vs pseudotime, fitted with LOESS regression lines.

Figure S3

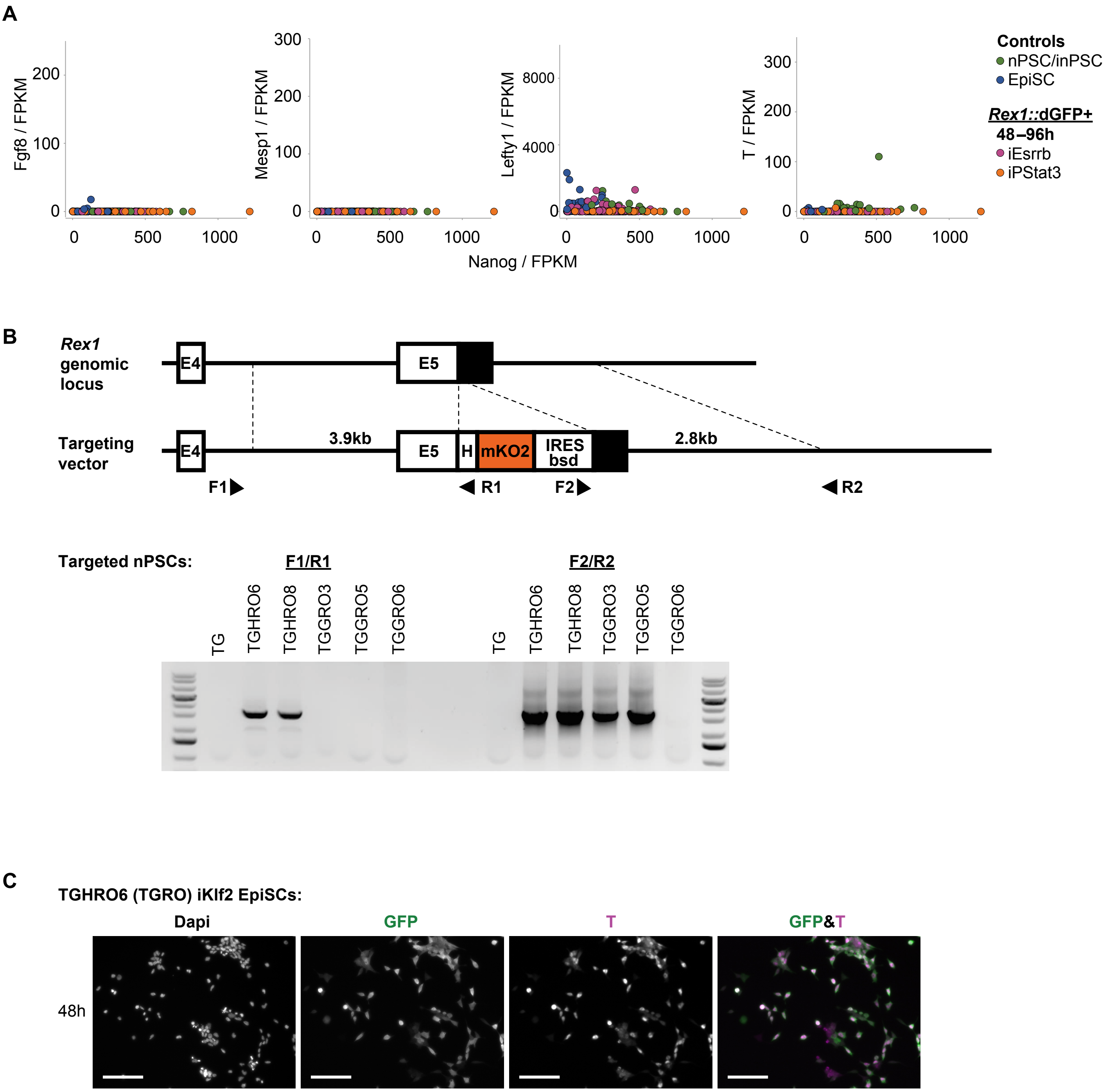
A
Fgf8 / FPKM
Mesp1 / FPKM
Lefty1 / FPKM
T / FPKM
Nanog / FPKM
Controls
nPSC/inPSC
EpiSC
Rex1::dGFP+
48–96h
iEsrrb
iPStat3
B
Rex1 genomic locus
Targeting vector
E4
E5
H
mKO2
IRES bsd
3.9kb
2.8kb
F1
R1
F2
R2
Targeted nPSCs:
F1/R1
F2/R2
TG
TGHRO6
TGHRO8
TGGRO3
TGGRO5
TGGRO6
C
TGHRO6 (TGRO) iKlf2 EpiSCs:
Dapi
GFP
T
GFP&T
48h

Figure S3, related to Figure 3:
iKlf2 reprogramming proceeds via a mesoderm-like state

(A) Scatter plots of mesodermal markers (Fgf8, Mesp1, Lefty1, T) against Nanog expression in iEsrrb and iPStat3 reprogramming intermediates, EpiSC (start) and nPSC/inPSC (end) identity controls. The *y*-axes are to the same scales as Fig 3A to facilitate comparison.

(B) Upper: Knock-in strategy for the *Rex1*::mKO2 fusion cassette, which was constructed by replacing the dGFP cassette of the *Rex1*::dGFP targeting vector (Kalkan et al., 2017). E4/5 = exon 4/5; H = helical linker (Arai et al., 2001); mKO2 = monomeric Kusabira Orange 2; bsd confers resistance to blasticidin if *Rex1* is expressed. Lower: resulting bsd-resistant clones were genotyped by PCR using the indicated F1/R1 or F2/R2 primers. TG is the $T^{+/GFP}$ parental nPSC line (Fehling et al., 2003). TGHRO are targeted with the helical linker (H) construct whereas TGGRO clones contained a glycine-serine linker. Correct targeting was obtained in nPSC clones TGHRO6&8, and TGHRO6 was used for subsequent experiments (simply denoted as TGRO in main figure panels).

(C) Immunofluorescent staining against GFP and T proteins, following induction of iKlf2 in TGHRO6 (TGRO) EpiSCs. Scale bars: 100µm.

Figure S4

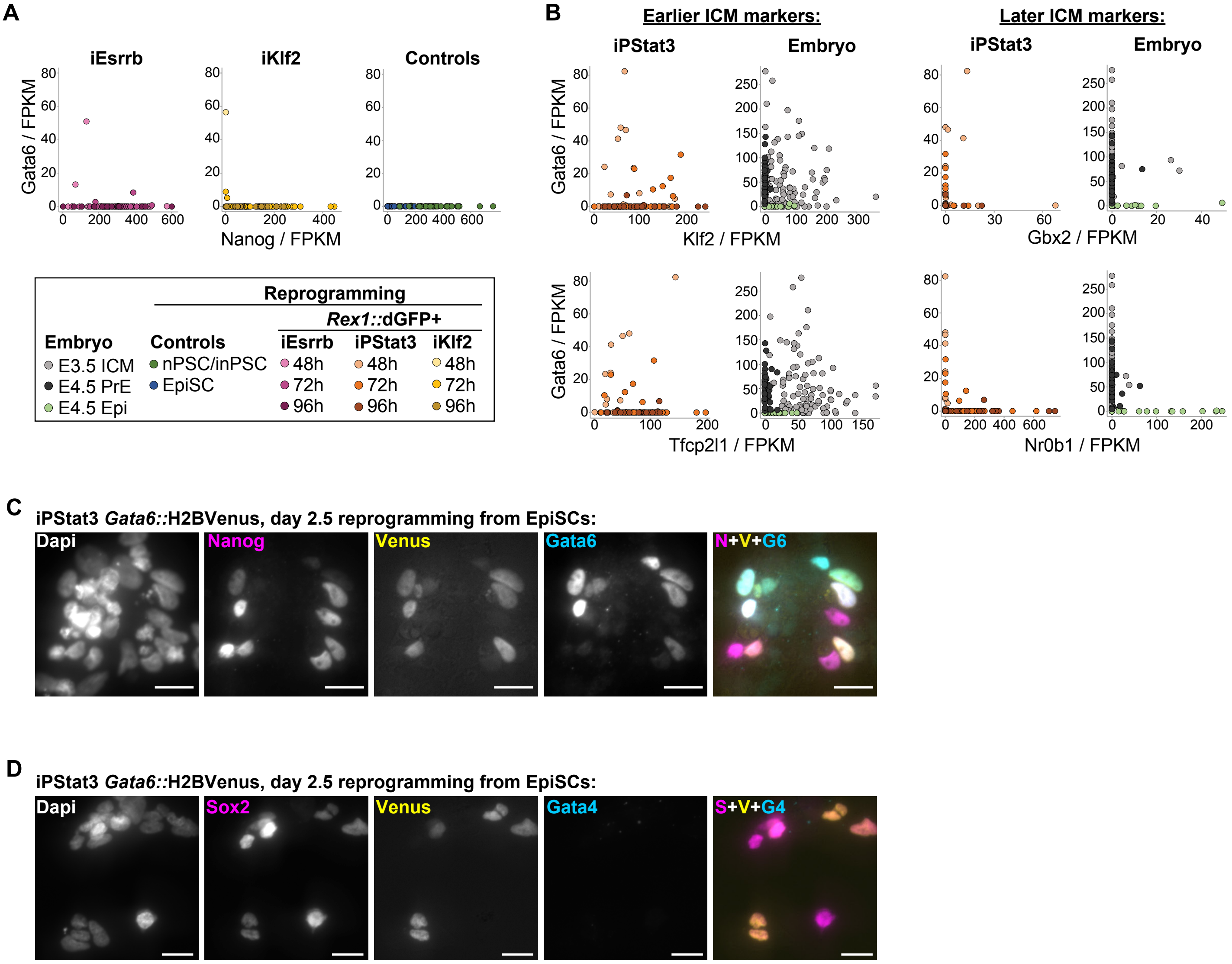
A
iEsrrb
iKlf2
Controls
Gata6 / FPKM
Nanog / FPKM
Reprogramming
Rex1::dGFP+
Embryo
E3.5 ICM
E4.5 PrE
E4.5 Epi
Controls
nPSC/inPSC
EpiSC
iEsrrb
iPStat3
iKlf2
48h
72h
96h
B
Earlier ICM markers:
Later ICM markers:
iPStat3
Embryo
Klf2 / FPKM
Gbx2 / FPKM
Tfcp2l1 / FPKM
Nr0b1 / FPKM
C
iPStat3 Gata6::H2BVenus, day 2.5 reprogramming from EpiSCs:
Dapi
Nanog
Venus
Gata6
N+V+G6
D
iPStat3 Gata6::H2BVenus, day 2.5 reprogramming from EpiSCs:
Dapi
Sox2
Venus
Gata4
S+V+G4

Figure S4, related to Figure 4:
iPStat3 reprogramming proceeds via an early ICM-like state

(A) Scatter plots of Gata6 vs Nanog expression in iEsrrb and iKlf2 reprogramming intermediates, EpiSC (start) and nPSC/inPSC (end) identity controls. The *y*-axes are to the same scales as Fig 4C to facilitate comparison.

(B) Scatter plots of Gata6 vs earlier or later ICM markers in iPStat3 reprogramming intermediates, E3.5 and E4.5 embryos. ICM: inner cell mass. Epi: epiblast. PrE: primitive endoderm. There is a temporal sequence of naïve gene activation in the embryo (Boroviak et al., 2015). Earlier markers (Klf2, Tfcp2l1) are expressed in Nanog+Gata6+ early ICM. In contrast, later markers (Gbx2, Nr0b1) are not activated until after Nanog+Gata6- naïve epiblast has segregated from Nanog-Gata6+ primitive endoderm. iPStat3 reprogramming intermediates emulate this *in vivo* progression: Klf2 and Tfcp2l1 are turned on earlier and are co-expressed with Gata6, whereas Gbx2 and Nr0b1 are activated later in Gata6- cells.

(C) iPStat3 was induced to reprogram *Gata6::*H2BVenus EpiSCs. Immunofluorescent staining against Gata6 and Nanog was performed at day 2.5. H2BVenus signal persisted without need for counterstaining. Scale bars: 20µm.

(D) Immunofluorescent staining against Gata4 and Sox2, 2.5 days after iPStat3 reprogramming induction, i.e. at the timepoint of sorting for injection. Scale bars: 20µm.

Figure S5

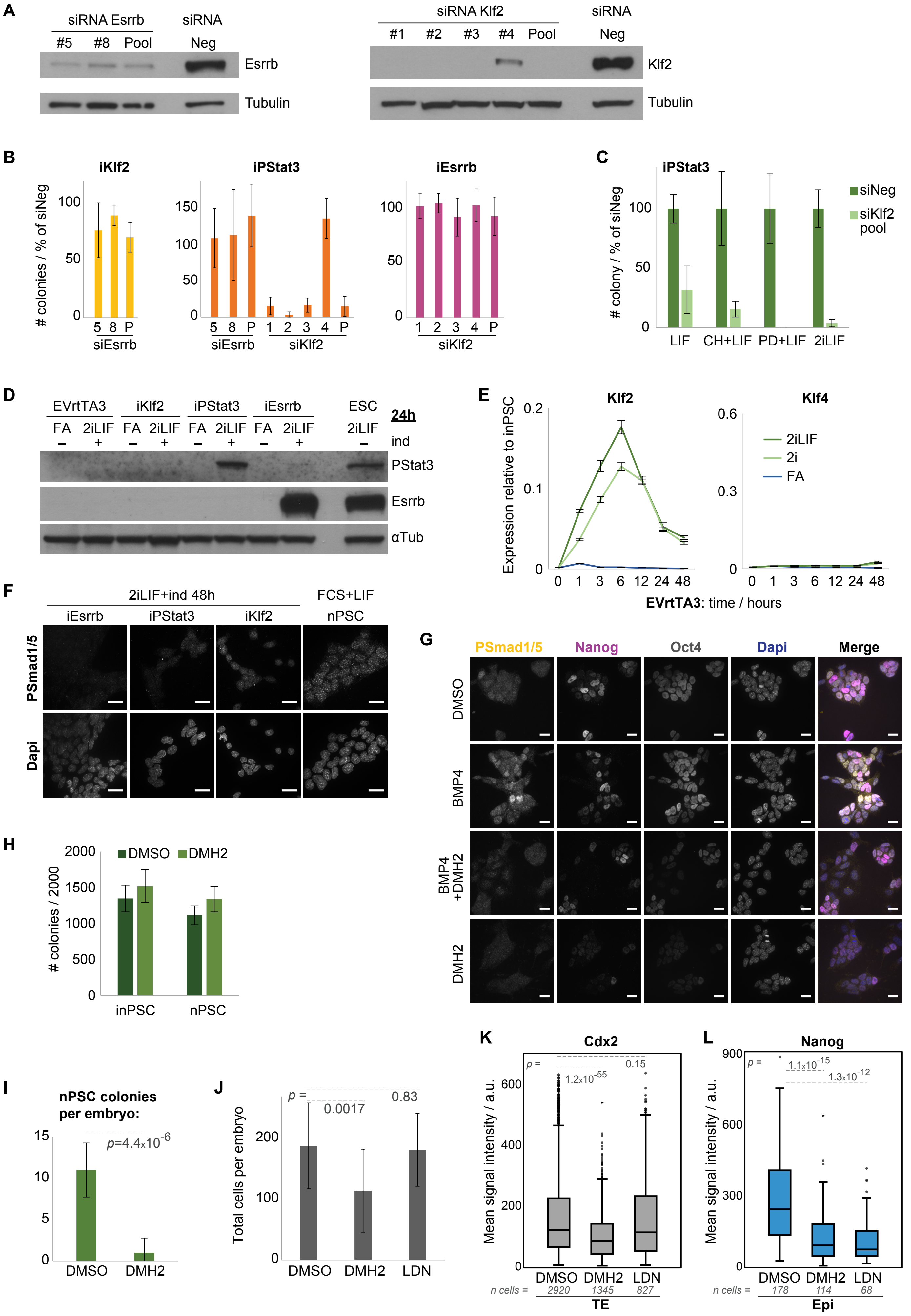
A
siRNA Esrrb
#5 #8 Pool
siRNA Neg
Esrrb
Tubulin
siRNA Klf2
#1 #2 #3 #4 Pool
siRNA Neg
Klf2
Tubulin
B
iKlf2
iPStat3
iEsrrb
colonies / % of siNeg
siEsrrb
siKlf2
C
iPStat3
colony / % of siNeg
siNeg
siKlf2 pool
LIF CH+LIF PD+LIF 2iLIF
D
EVrtTA3 iKlf2 iPStat3 iEsrrb ESC
FA 2iLIF FA 2iLIF FA 2iLIF FA 2iLIF 2iLIF
24h
ind
PStat3
Esrrb
αTub
E
Klf2
Klf4
Expression relative to inPSC
2iLIF
2i
FA
EVrtTA3: time / hours
F
2iLIF+ind 48h
FCS+LIF
iEsrrb iPStat3 iKlf2 nPSC
PSmad1/5
Dapi
G
PSmad1/5 Nanog Oct4 Dapi Merge
DMSO
BMP4
BMP4 +DMH2
DMH2
H
DMSO DMH2
colonies / 2000
inPSC nPSC
I
nPSC colonies per embryo:
p=4.4x10^-6
DMSO DMH2
J
Total cells per embryo
p = 0.0017 0.83
DMSO DMH2 LDN
K
Cdx2
Mean signal intensity / a.u.
p = 1.2x10^-55 0.15
DMSO DMH2 LDN
n cells = 2920 1345 827
TE
L
Nanog
Mean signal intensity / a.u.
p = 1.1x10^-15 1.3x10^-12
DMSO DMH2 LDN
n cells = 178 114 68
Epi

Figure S5, related to Figure 5:
Routes have distinct genetic and signal requirements

(A) Western blots against Esrrb and Klf2, after siRNA treatment of nPSCs in 2iLIF. αTubulin provides loading control. siRNAs were applied from 0–16h, and samples harvested at 48h. Individual siRNAs were used at 10µM, whereas pools were comprised of 4x 2.5µM.

(B) siRNA treatment was performed in a single 16h pulse at reprogramming onset, with individual 10µM siRNAs as well as pools (P) of 4x 2.5µM. Reprogramming was induced with 2iLIF+dox (iEsrrb, iKlf2) or 2iLIF+GCSF (iPStat3). Selection was performed from day 4 with 2iLIF+blasticidin and inPSC colonies were scored at day 8, presented as mean ± SD (n=6) relative to siNeg. Pools of 4 siRNAs, each at a quarter concentration and targeting a different region of the mRNA, are expected to minimise off-target effects. With the exception of inefficient Klf2 siRNA #4 (Fig S5A), individual siRNAs give the same outcomes as pools: iPStat3-driven reprogramming is dependent on early Klf2 expression, whereas iEsrrb is not (Fig 5B); Esrrb KD at reprogramming onset does not abolish reprogramming by any driver. This is consistent with lack of Esrrb expression by any other driver at 24h (Fig 5A, S5D).

(C) Klf2 KD was performed at iPStat3-driven reprogramming onset with a single 16h pulse of siRNA pool, in the indicated conditions +GCSF from day 0–4, then inPSC colonies were selected from day 4–8 in 2iLIF+blasticidin. inPSC colonies were scored on day 8, presented as mean ±SD (n=3) relative to siNeg. PD=PD03; CH=Chiron; 2i=PD+CH. iPStat3 sensitivity to Klf2 KD is alleviated in the absence of PD03, reminiscent of the ability to rescue embryo-derived *Nanog*$^{-/-}$ EpiSC reprogramming in Chiron+LIF but not 2iLIF (Stuart et al., 2014). Notably, both Nanog and Klf2 are considered targets of PD03 (Silva et al., 2009; Yeo et al., 2014): it appears they are required to transduce its positive input to the naïve network and, in their absence, PD03 is actively detrimental to reprogramming. Hence, interplay between TFs and signals can fundamentally modulate each other's role in identity specification.

(D) Western blots against PStat3 and Esrrb, after 24h in FA or 2iLIF+driver induction (ind: GCSF for iPStat3, dox for others). αTubulin provides loading control.

(E) RT-qPCR analysis of EVrtTA3 EpiSCs in the indicated condition +dox. Mean gene expression is displayed relative to Gapdh and normalised to inPSCs, ±SD (n=3). The axes are to the same scales as Fig 5F to facilitate comparison.

(F) Immunofluorescent staining 48h after reprogramming induction (ind: GCSF for iPStat3, dox for others). nPSCs cultured in FCS+LIF provide a positive control for PSmad1/5. Maximum intensity projections of Z-stack slices are presented. Scale bars: 20µm.

(G) Validation of 3µM DMH2 efficacy. Wild-type nPSCs cultured in FCS+LIF were treated for 24h as indicated. BMP signalling is known to be active and important for pluripotency maintenance in FCS+LIF (Ying et al., 2003), unlike in 2iLIF. Maximum intensity projections of Z-stack slices are presented. Scale bars: 20µm.

(H) 3µM DMH2 or 1:1000 DMSO was applied to nPSCs or previously established iKlf2 inPSCs, at clonal density in 2iLIF. Naïve colonies were scored after 4 days, presented as mean ±SD (n=3).

(I–K) Mouse blastocysts were cultured with DMSO or BMP signalling inhibitor from cavitation onset until the late blastocyst stage, by which point the epiblast (Epi) and primitive endoderm (PrE) lineages are fully segregated, thus allowing us to study the impact on each lineage.

(I) Quantitative nPSC-derivation was performed from late blastocysts, following treatment with either DMSO or 3µM DMH2. Immunosurgery was performed to remove the trophectoderm (TE), then the inner cell mass (ICM, comprising Epi+PrE) was dissociated to single cells. 10 single cells were manually transferred to each 96well and cultured in feeder-free 2iLIF conditions, without any further DMSO/DMH2 treatment so that we could assess whether the Epi of the embryo was already affected. By plating 10 single cells per well, we could measure absolute nPSC-derivation efficiency independently of embryo size. The number of nPSC

colonies was scored on day 6, and is presented per 10 single cells (main Fig 5K) and as mean per embryo ±SD (here). DMSO n=7; DMH2 n=8. nPSC-identity was subsequently confirmed by RT-qPCR (data not shown).

(J) Mean number of total cells per embryo ± SD. DMSO n=23, DMH2 n=18, LDN n=7 embryos. Note that the lower cell number per DMH2-treated embryo was not due to developmental retardation: Epi and PrE endoderm lineages segregated, the proportion of cells per lineage was unchanged (Fig 5L), and expression of Gata4 indicates late PrE (Artus et al., 2011).

(K) Late blastocysts were fixed and stained for Cdx2, Gata4 and Oct4, following treatment with 1:1000 DMSO, 3µM DMH2 or 0.3µM LDN. Mean signal intensity was quantified for each nucleus, and is presented as box-and-whisker plots for Cdx2 in TE cells (DMSO n=23, DMH2 n=18, LDN n=7 embryos). Oct4 and Gata4 results are presented in main Fig 5N.

(L) A subset of embryos was also stained for Nanog. Mean signal intensity was quantified for each nucleus, and is presented as box-and-whisker plots for Nanog in Epi cells (DMSO n=9, DMH2 n=11, LDN n=4).

Figure S6

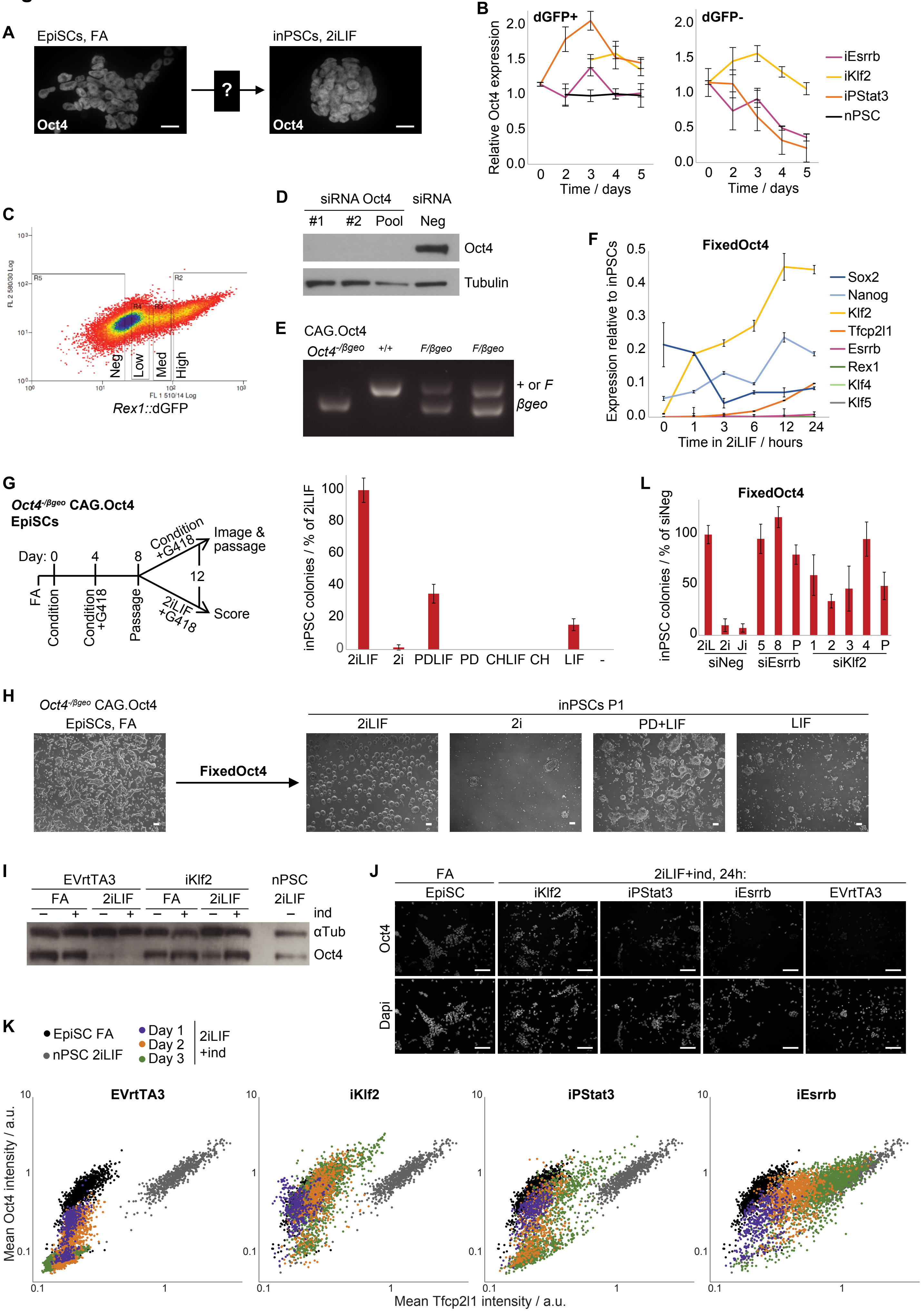

A
EpiSCs, FA
inPSCs, 2iLIF
Oct4
Oct4
?
B
dGFP+
dGFP-
Relative Oct4 expression
Time / days
iEsrrb
iKlf2
iPStat3
nPSC
C
Rex1::dGFP
Neg
Low
Med
High
D
siRNA Oct4
siRNA
#1
#2
Pool
Neg
Oct4
Tubulin
E
CAG.Oct4
Oct4-/βgeo
+/+
F/βgeo
F/βgeo
+ or F
βgeo
F
FixedOct4
Expression relative to inPSCs
Sox2
Nanog
Klf2
Tfcp2l1
Esrrb
Rex1
Klf4
Klf5
Time in 2iLIF / hours
G
Oct4-/βgeo CAG.Oct4
EpiSCs
Day: 0
4
8
12
FA
Condition
Condition +G418
Passage
Condition +G418
Image & passage
2iLIF +G418
Score
inPSC colonies / % of 2iLIF
2iLIF
2i
PDLIF
PD
CHLIF
CH
LIF
-
L
FixedOct4
inPSC colonies / % of siNeg
2iL
2i
Ji
5
8
P
1
2
3
4
P
siNeg
siEsrrb
siKlf2
H
Oct4-/βgeo CAG.Oct4
EpiSCs, FA
FixedOct4
inPSCs P1
2iLIF
2i
PD+LIF
LIF
I
EVrtTA3
iKlf2
nPSC
FA
2iLIF
FA
2iLIF
2iLIF
ind
αTub
Oct4
J
FA
2iLIF+ind, 24h:
EpiSC
iKlf2
iPStat3
iEsrrb
EVrtTA3
Oct4
Dapi
K
EpiSC FA
nPSC 2iLIF
Day 1
Day 2
Day 3
2iLIF +ind
EVrtTA3
iKlf2
iPStat3
iEsrrb
Mean Oct4 intensity / a.u.
Mean Tfcp2l1 intensity / a.u.

Figure S6, related to Figure 6:
EpiSC reprogramming converges on the fine-tuning of Oct4 expression

(A) Immunofluorescent staining of Oct4 expression in *Rex1::*dGFP EpiSCs and inPSCs. Maximum intensity projections of Z-stack slices are presented. Scale bars: 20µm.

(B) Timecourse RT-qPCR analyses of *Rex1::*dGFP+ and dGFP– EpiSC reprogramming intermediates in 2iLIF+dox/GCSF, and of *Rex1::*dGFP+ nPSCs in 2iLIF. Mean Oct4 expression is displayed relative to Gapdh and normalised to nPSC day 2, ±SD (n=3).

(C) FACS-plot showing *Rex1::*dGFP levels during reprogramming. The example here is iEsrrb 2iLIF+dox day 3. *Rex1::*dGFP high, medium (med), low, and negative (neg) gates are indicated. *Rex1::*dGFP negative and high gates were set according to *Rex1::*dGFP EpiSCs and nPSCs respectively. Low and medium gates subdivide the intervening levels.

(D) Western blot against Oct4, after siRNA treatment of nPSCs in 2iLIF. αTubulin provides loading control. siRNAs were applied from 0–16h, and samples harvested at 48h. Individual siRNAs were used at 10µM, whereas pool was comprised of 4x 2.5µM. Whilst other genetic requirements at reprogramming onset are driver-specific (Fig S5B), Oct4 KD at reprogramming onset abolished reprogramming for iKlf2, iEsrrb and iPStat3 (Fig 6F).

(E) Genotyping of $Oct4^{-/\beta geo}$ CAG.Oct4 EpiSCs (FixedOct4), with $Oct4^{+/+}$ and $Oct4^{F/\beta geo}$ controls. *F*=flox allele. The *βgeo* allele confers resistance to G418 if *Oct4* promoter is active.

(F) Gene expression analyses by RT-qPCR of FixedOct4 EpiSCs following reprogramming induction by 2iLIF. Mean gene expression is displayed relative to Gapdh and normalised to inPSCs, ±SD (n=3). The initial transcriptional response of FixedOct4 to 2iLIF has features in common with each of the other drivers: rapid upregulation of Klf2 (as also observed for iPStat3 & iEsrrb); moderate upregulation of Tfcp2l1 (iPStat3 & iEsrrb); poor upregulation of Klf4 (iPStat3 & iKlf2); poor upregulation of Klf5 (iEsrrb & iKlf2); poor upregulation of iEsrrb (iPStat3 & iKlf2). Thus, besides the Klf5 discrepancy, FixedOct4 initiation is most similar to iPStat3, consistent with its reprogramming impetus coming from the environment including LIF.

(G–H) $Oct4^{-/\beta geo}$ CAG.Oct4 EpiSCs were plated at 2000/24well in FA. The following day, medium was changed to N2B27 ± Chiron ± PD03 (PD) ± LIF in all permutations (n=3). After 4 days, G418 was applied to select for endogenous *Oct4* promoter activity. Quantification of reprogramming efficiency was inappropriate at this point, since some conditions are permissive compared to 2iLIF. Instead, on day 8, each 24well was passaged in its entirety to 2 x 6wells, one maintaining the condition +G418 and one swapped to 2iLIF+G418 to challenge inPSC clonogenicity in this naïve-selective condition. P1 inPSC colonies were scored on day 12 in 2iLIF+G418, presented as mean ± SD (n=3) **(G).** Condition +G418 phase images are shown for those conditions which successfully generated inPSCs **(H)**. Scale bars: 100µm. We found that FixedOct4 +LIF was the minimal requirement for naïve pluripotency specification, with derivative inPSCs expandable in LIF+G418 or 2iLIF+G418 for at least 8 passages. Curiously, Chiron+LIF did not instruct naïve pluripotency acquisition for FixedOct4 EpiSCs despite initial emergence of naïve-like morphology.

(I) Western blot against Oct4 in EVrtTA3 and iKlf2 EpiSCs, after 24h in FA or 2iLIF ± induction (ind) with dox. αTubulin provides loading control.

(J) Immunofluorescent staining of Oct4 expression in *Rex1::*dGFP iKlf2, iPStat3, iEsrrb and EVrtTA3 EpiSCs, after 24h in 2iLIF+induction (ind: GCSF for iPStat3, dox for others). Scale bars: 100µm.

(K) Quantification of Oct4 immunofluorescent staining during EpiSC reprogramming, on a total of 14,736 single cells. Samples included all cells and were not sorted according to *Rex1::*dGFP reporter, i.e. productive and unproductive cells are present to capture all events unbiasedly. Tfcp2l1 co-staining indicates progression towards the naïve pluripotent identity. Oct4 protein is lost in negative control EVrtTA3 EpiSCs following medium switch, but this can be rescued by the reprogramming drivers. Oct4 is maintained on the protein level in cells progressing

towards naïve pluripotency. Conversely, there is no evidence of naïve acquisition if Oct4 protein is lost. EpiSC and nPSC controls are shared between plots.

(L) To address the role of endogenous Klf2, Esrrb and PStat3 during FixedOct4 EpiSC reprogramming, Klf2 and Esrrb KD were performed at reprogramming onset with a single 16h pulse of individual (10µM) or pooled (4x 2.5µM) siRNAs. Reprogramming was induced with 2iLIF, selection was performed from day 4 with 2iLIF+G418, then inPSC colonies were scored at day 8, presented as mean ± SD (n=6) relative to siNeg. To address the role of PStat3, we instead conducted experiments in the absence of LIF (L) and/or in the presence of Jak inhibitor (Ji), to ensure that Stat3 was not activated. Overall, LIF/Stat3 signal inhibition had the greatest impact on FixedOct4 reprogramming, whilst Esrrb KD had the least.

Figure S7

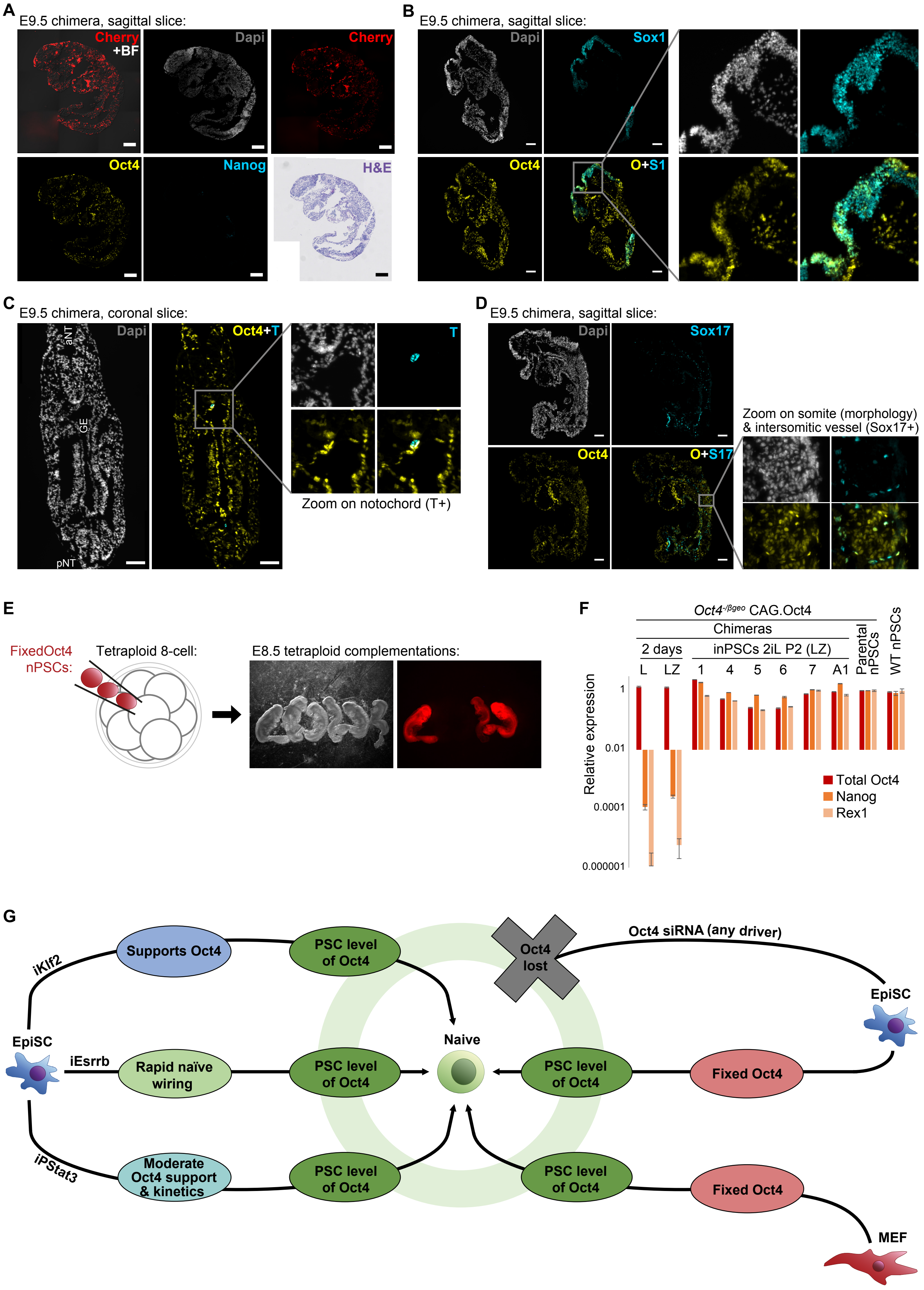
A
E9.5 chimera, sagittal slice:
Cherry +BF
Dapi
Cherry
Oct4
Nanog
H&E
B
E9.5 chimera, sagittal slice:
Dapi
Sox1
Oct4
O+S1
C
E9.5 chimera, coronal slice:
aNT
GE
pNT
Dapi
Oct4+T
T
Zoom on notochord (T+)
D
E9.5 chimera, sagittal slice:
Dapi
Sox17
Oct4
O+S17
Zoom on somite (morphology) & intersomitic vessel (Sox17+)
E
FixedOct4 nPSCs:
Tetraploid 8-cell:
E8.5 tetraploid complementations:
F
Oct4-/βgeo CAG.Oct4
Chimeras
2 days
inPSCs 2iL P2 (LZ)
L
LZ
1
4
5
6
7
A1
Parental nPSCs
WT nPSCs
Relative expression
1
0.01
0.0001
0.000001
Total Oct4
Nanog
Rex1
G
Supports Oct4
PSC level of Oct4
Oct4 lost
Oct4 siRNA (any driver)
iKlf2
EpiSC
EpiSC
iEsrrb
Rapid naïve wiring
PSC level of Oct4
Naive
PSC level of Oct4
Fixed Oct4
iPStat3
Moderate Oct4 support & kinetics
PSC level of Oct4
PSC level of Oct4
Fixed Oct4
MEF

Figure S7, related to Figure 7:
PSC-level of Oct4 is sufficient for somatic cell reprogramming

(A–D) Differentiation of FixedOct4 cells in E9.5 chimeras was analysed by immunostaining of 8µm cryosections, taken in various sectioning planes. This extensively demonstrates bonafide contribution of FixedOct4 cells to downstream lineages in the embryo, as evidenced by continued Oct4 expression together with appropriate lineage markers.

(A) Cherry and brightfield (BF) images were acquired on rehydrated sagittal slice, prior to immunofluorescent staining against Oct4 and Nanog. Contribution of FixedOct4 cells was high and widespread, visualised by Cherry signal from the CAG.Oct4.2A.Cherry transgene. Importantly, Cherry signal agreed with Oct4 counter-stain, whereas Nanog expression was not detected. This confirms that the cells had exited pluripotency yet maintained transgenic Oct4 expression. Subsequently, H&E staining was performed on the same slice. Contribution of FixedOct4 cells to various tissues is evident from H&E histological analyses. Scale bars: 200µm.

(B) Immunofluorescent staining against Oct4 and Sox1 on sagittal slice (midline section). Zoom of the indicated region shows the developing brain. Scale bars: 100µm.

(C) Immunofluorescent staining against Oct4 and T on coronal slice. Zoom of the indicated region shows the notochord. Scale bars: 100µm. GE=gut endoderm; NT=neural tube; a=anterior; p=posterior.

(D) Immunofluorescent staining against Oct4 and Sox17 on a sagittal slice. Zoom of the indicated region shows intersomitic blood vessels. Scale bars: 100µm.

(E) Tetraploid C57BL/6 embryos were generated by cell fusion at the 2-cell stage, then cultured to the 8-cell stage. *Oct4*$^{-/\beta geo}$ CAG.Oct4.2A.Cherry nPSCs were injected into tetraploid 8-cell embryos, then transferred to recipients. Resultant embryos were collected at E8.5. Phase and Cherry images are shown, of 3 tetraploid complementations and 3 stage-matched wild-type embryos from a different litter. Scale bars: 100µm. This shows that FixedOct4 cells are capable of performing tetraploid complementation, a stringent assay for developmental contribution.

(F) Differentiated FixedOct4 cells were derived from E9.5 chimeras, then reprogrammed in LIFaza (LZ) or LIF only (L) from day 0–6, followed by 2iLIF until day 10 (Fig 7C). RT-qPCR expression analyses are shown after two days of treatment with L or LZ on cells from chimera 1, for inPSCs at passage 2 in 2iLIF+G418 after derivation in LZ then 2iLIF from chimeras 1–7, and after derivation directly in 2iLIF for a chimeric allantois (A1). Mean expressions ± SD (2 technical replicates per embryo) are presented relative to Gapdh then normalised to parental nPSCs.

(G) Schematic summarising the unifying, required and sufficient feature of correct Oct4 level, which permits cells to transit into naïve pluripotency. Left: diverse logics by which different EpiSC reprogramming drivers achieve correct Oct4 expression. When control EpiSCs are exposed to naïve signals, Oct4 expression drops as the primed network is disrupted. Reprogramming drivers must actively overcome this in order to undergo the identity transition. Although iKlf2, iPStat3 and iEsrrb drive reprogramming by transcriptionally and mechanistically distinct routes (Fig 6A), all ultimately achieve the convergent feature of correct Oct4 level and thus can reach the same naïve pluripotent destination. Right: the functional importance of precise Oct4 expression was confirmed by transient KD, which abolishes reprogramming by all drivers. Conversely, fixing Oct4 to PSC level is sufficient for reprogramming under only signal instruction, from EpiSCs and from developmentally more advanced cell types including MEFs. Therefore, appropriate Oct4 expression is the pivotal feature for transition into the naïve pluripotent identity, regardless of the route of approach.

Supplemental Tables

**GO biological process**	***p* value**
supramolecular fiber organization (GO:0097435)	4.66E-08
cytoskeleton organization (GO:0007010)	3.89E-06
regulation of cellular component movement (GO:0051270)	4.52E-06
cellular component organization (GO:0016043)	5.00E-06
cellular component organization or biogenesis (GO:0071840)	2.56E-05
regulation of actin filament-based process (GO:0032970)	2.93E-05
regulation of localization (GO:0032879)	5.97E-05
regulation of biological quality (GO:0065008)	6.72E-05
regulation of multicellular organismal process (GO:0051239)	1.08E-04
regulation of supramolecular fiber organization (GO:1902903)	1.48E-04
regulation of cell motility (GO:2000145)	4.25E-04
actin cytoskeleton organization (GO:0030036)	4.72E-04
regulation of cell migration (GO:0030334)	5.01E-04
regulation of locomotion (GO:0040012)	6.52E-04
actin filament organization (GO:0007015)	7.41E-04
regulation of developmental process (GO:0050793)	1.04E-03
negative regulation of biological process (GO:0048519)	1.75E-03
anatomical structure development (GO:0048856)	1.99E-03
actin filament-based process (GO:0030029)	2.92E-03
negative regulation of cellular process (GO:0048523)	3.46E-03
regulation of system process (GO:0044057)	3.81E-03
protein localization to plasma membrane (GO:0072659)	4.32E-03
developmental process (GO:0032502)	5.19E-03
regulation of cell differentiation (GO:0045595)	5.80E-03
cellular process (GO:0009987)	8.64E-03
system development (GO:0048731)	9.78E-03
regulation of cytoskeleton organization (GO:0051493)	1.51E-02
regulation of actin cytoskeleton organization (GO:0032956)	1.84E-02
regulation of biological process (GO:0050789)	1.96E-02
regulation of cellular component organization (GO:0051128)	1.99E-02
animal organ development (GO:0048513)	2.90E-02
biological regulation (GO:0065007)	3.62E-02
regulation of cell population proliferation (GO:0042127)	4.60E-02

Table S1, related to Figure 2:
Gene ontology enrichment for genes contributing to initial iKlf2 diversion

Gene ontology (GO) analysis for biological processes enriched in the gene list contributing to –PC1 dimension on the iKlf2 PCA plot (main Fig 2D left panel) (contribution score <–0.5). GO analysis was conducted using the PANTHER Overrepresentation Test with Bonferroni correction, and processes with $p < 0.05$ are presented in the table above.

PROBE	SOURCE	IDENTIFIER
Gapdh VIC-labelled TaqMan probe	Applied Biosystems	Cat#4352339E
Esrrb FAM-labelled TaqMan probe	Applied Biosystems	ID#Mm00442411_m1
Fgf5 FAM-labelled TaqMan probe	Applied Biosystems	ID#Mm00438918_m1
Gata6 FAM-labelled TaqMan probe	Applied Biosystems	ID#Mm00802636_m1
Klf2 FAM-labelled TaqMan probe	Applied Biosystems	ID#Mm01244979_g1
Klf4 FAM-labelled TaqMan probe	Applied Biosystems	ID#Mm00516104_m1
Klf5 FAM-labelled TaqMan probe	Applied Biosystems	ID#Mm00456521_m1
Nanog FAM-labelled TaqMan probe	Applied Biosystems	ID#Mm02384862_g1
Pou5f1 FAM-labelled TaqMan probe	Applied Biosystems	ID#Mm00658129_gH
Sox2 FAM-labelled TaqMan probe	Applied Biosystems	ID#Mm03053810_s1
T (Brachyury) FAM-labelled TaqMan probe	Applied Biosystems	ID#Mm01318252_m1
Tfcp2l1 FAM-labelled TaqMan probe	Applied Biosystems	ID#Mm00470119_m1
Zfp42 FAM-labelled TaqMan probe	Applied Biosystems	ID#Mm03053975_g1

Table S2, related to Key Resource Table:
RT-qPCR Taqman probes

PRIMER	SOURCE	IDENTIFIER
SYBR RT-qCPR primer for Col1a1 F: GATCTGTATCTGCCACAATG	Sigma-Aldrich	Cat#KSPQ12012G_ID: 8812036114-10/0
SYBR RT-qCPR primer for Col1a1 R: TGGTGATACGTATTCTTCCG	Sigma-Aldrich	Cat#KSPQ12012G_ID: 8812036114-10/1
SYBR RT-qCPR primer for Prrx1 F: GAAAAAGAACTTCTCCGTCAG	Sigma-Aldrich	Cat#KSPQ12012G_ID: 8812036114-30/0
SYBR RT-qCPR primer for Prrx1 R: CTTTCTCTTCTTCTTCTCCTC	Sigma-Aldrich	Cat#KSPQ12012G_ID: 8812036114-30/1
SYBR RT-qCPR primer for Gapdh F: CCCACTAACATCAAATGGGG	Sigma-Aldrich	Custom
SYBR RT-qCPR primer for Gapdh R: CCTTCCACAATGCCAAAGTT	Sigma-Aldrich	Custom
Primer for Oct4 genotyping (wt and flox): GAGCTTATGATCTGATGTCCATCTCTGTGC	Le Bin et al., 2014	N/A
Primer for Oct4 genotyping (βgeo): GGGCTGACCGCTTCCTCGTGCTTTACG	Le Bin et al., 2014	N/A
Primer for Oct4 genotyping (all): GCCTTCCTCTATAGGTTGGGCTCCAACC	Le Bin et al., 2014	N/A
Primer for Rex1-mKO2 targeting, F1: TCGTGTGACTCTGCATCTGT	This study	N/A
Primer for Rex1-mKO2 targeting, R1: CTGCCTCTTTAGCTGCGG	This study	N/A
Primer for Rex1-mKO2 targeting, F2: ATTCGTGAATTGCTGCCCTC	This study	N/A
Primer for Rex1-mKO2 targeting, R2: GAGGCAGAGGAACAGGACTT	This study	N/A

Table S3, related to Key Resource Table:
Primers
